# Supplementary material for: Enhanced CRISPR-based DNA demethylation by Casilio-ME-mediated RNA-guided coupling of methylcytosine oxidation and DNA repair pathways
Source: Nat Commun. 2019 Sep 20;10:4296. doi: 10.1038/s41467-019-12339-7 (PMC6754513; doi:10.1038/s41467-019-12339-7)
Supplement: Supplementary file 1 — Supplementary Information [file 41467_2019_12339_MOESM1_ESM.pdf]

Enhanced CRISPR-based DNA demethylation by Casilio-ME-mediated RNA-guided coupling of methylcytosine oxidation and DNA repair pathways

Supplementary Information

Taghbalout et al.

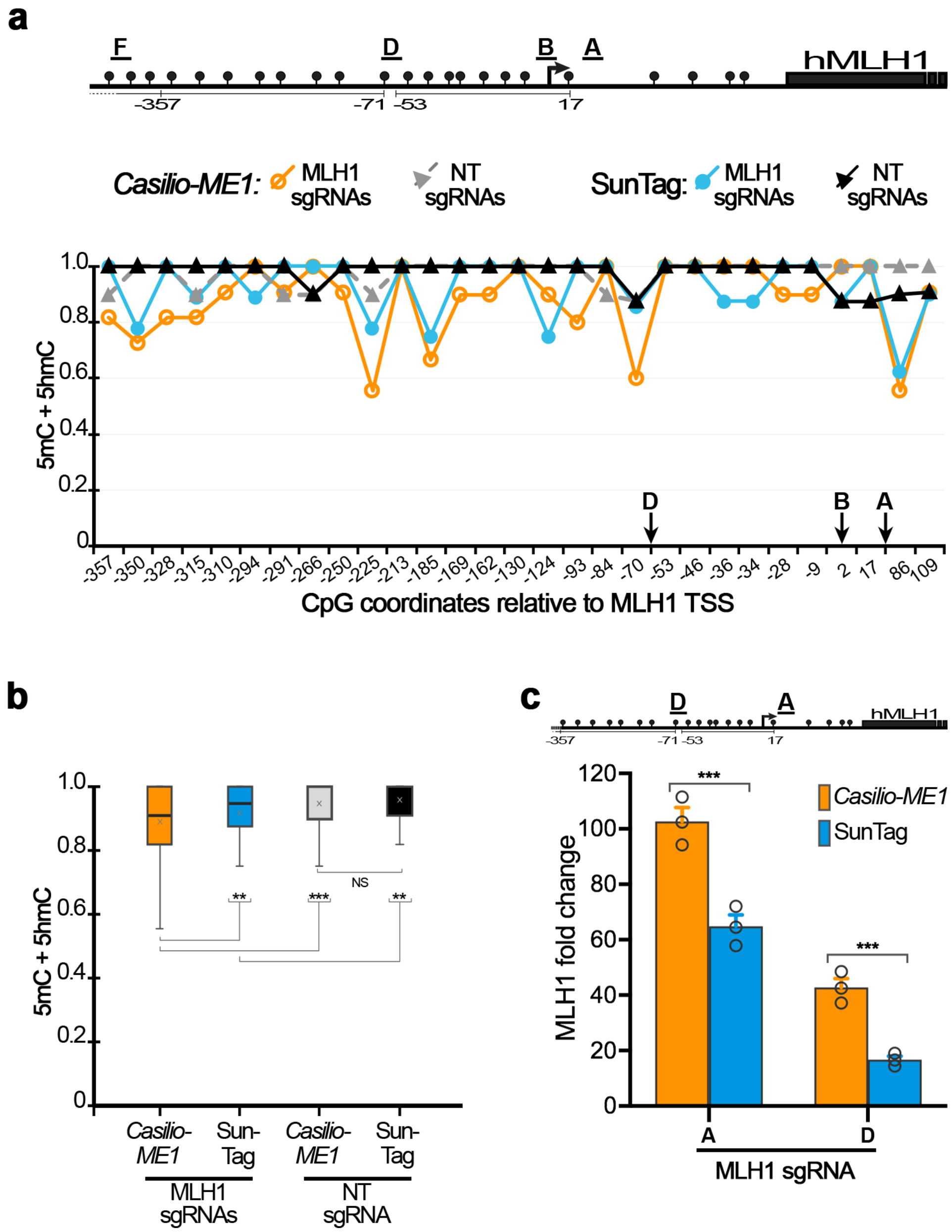

Supplementary Figure 1

***Casilio-ME1* and SunTag mediated 5mC demethylation. (a)** upper panel: *MLH1* promoter and associated CGI. CpGs (lollipops), TSS (arrow), and the sgRNAs used for targeting TET1 effectors (A, B, D, F) are shown. Lower panel: methylation frequency (5mC + 5hmC) at *MLH1* promoter regions of cells transfected with components of *Casilio-ME1* or SunTag in the presence of *MLH1*-sgRNAs or NT-sgRNA is shown. Methylation frequency from BSeq of cloned *MLH1* amplicons plotted against CpG relative positions, and CpGs that overlap *MLH1*-sgRNAs sequence targets (arrows) are shown. Statistical significance of difference in methylation frequencies obtained were tested. $p<0.05$, one-way ANOVA. **(b)** Box plot of methylation frequency from BSeq of cloned *MLH1* amplicons from transfected cells shown in (a). For each box plot the thick line inside the

box represents the median value and the surrounding bottom and top lines represent the 25th and 75th percentiles. The whiskers represent min and max values, the x represents the mean value. NS, not significant, $p>0.05$, ** $p<0.05$, and *** $p<0.001$, one-way ANOVA. **(c)** *MLH1* mRNA relative levels (mean fold change ± S.E.M.; n=3) in cells transfected with *Casilio-ME1* or SunTag components in the presence of one targeting sgRNA. Drawing above the column plot shows *MLH1* promoter region and the sgRNA used for targeting (A or D). *** $p<0.005$, one-way ANOVA.

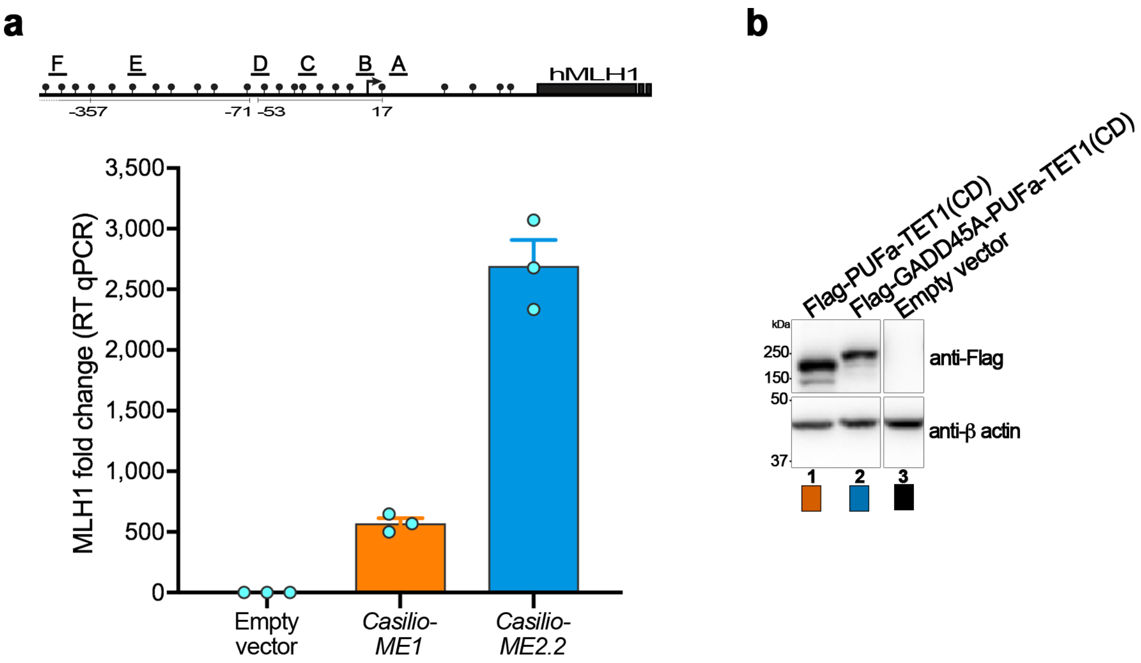

Supplementary Figure 2

Protein levels of TET1-effectors in *Casilio-ME1* or *Casilio-ME2.2* transfected cells. **(a)** *MLH1* mRNA relative levels (mean fold change ± S.E.M.; n=3) in cells transfected with components of Flag-tagged *Casilio-ME1* (Flag-PUFa-TET1(CD)) or *Casilio-ME2.2* (Flag-GADD45A-PUFa-TET1(CD)) in the presence of indicated *MLH1*-sgRNAs depicted above the plot. **(b)** Western blot analysis using anti-Flag, anti-β actin monoclonal antibodies and proteins extracted from cells analyzed (a). Proteins extracted from cells transfected with Flag-PUFa-TET1(CD) *Casilio-ME1* effector (lane 1), Flag-GADD45A-PUFa-TET1(CD) *Casilio-ME2.2* effector (lane 2), or empty vector (lane 3), and size of protein ladder in kDa are shown.

Supplementary Figure 3

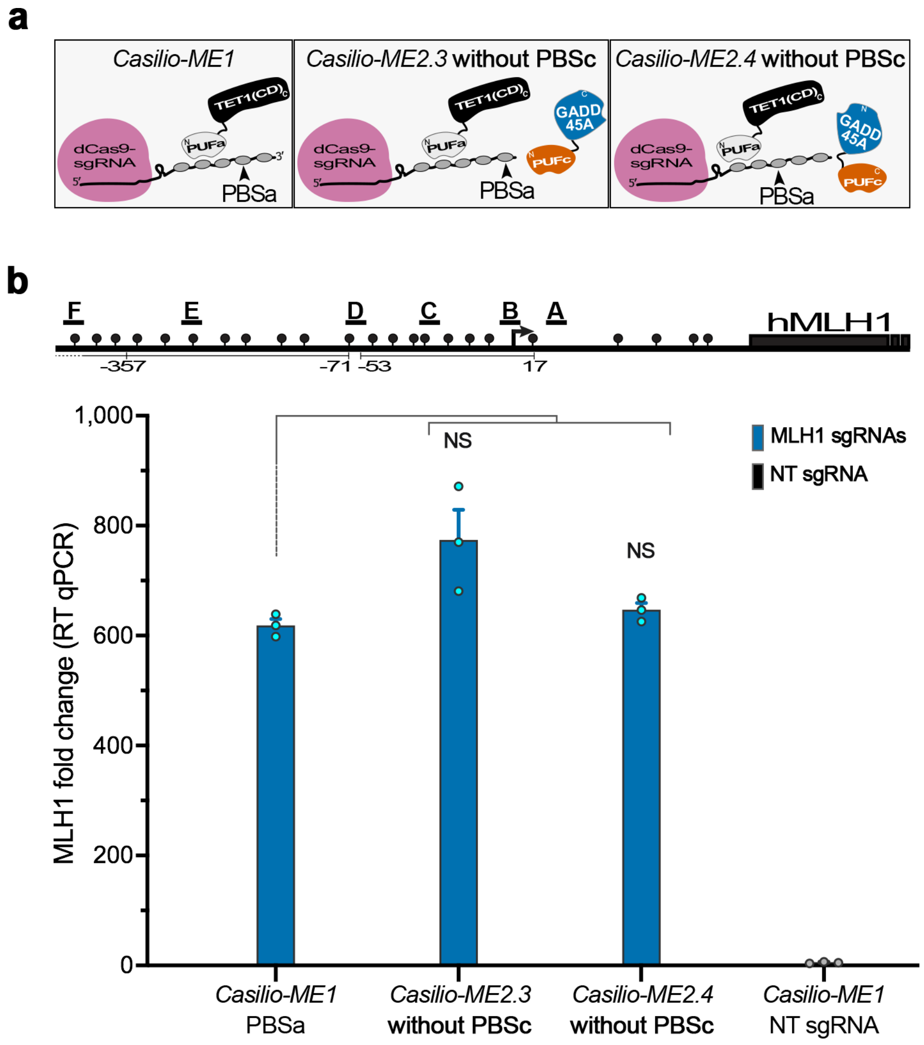

Supplementary Figure 3

Enhanced *MLH1* activation by *Casilio-ME2* platforms requires co-delivery of TET1(CD) and GADD45A to genomic sites. (a) Illustration of *Casilio-ME* platforms showing effector modules of PUFa and PUFc protein fusions used to transfect cells in the presence of NT-sgRNA or *MLH1*-sgRNAs containing PBSa but lacking PBSc required for targeting PUFc-based effectors to genomic sites. TET1(CD) (black), GADD45A (blue), PUFa (light grey), PUFc (orange), amino (N) and carboxyl (C) termini of protein fusions are arbitrarily shown. **(b)** *MLH1* mRNA relative levels (mean fold change ± S.E.M.; n=3) in cells transfected with the indicated components of *Casilio-ME1, Casilio-ME2.3, Casilio-ME2.4* in the presence of sgRNAs that comprised PBSa but lacked PBSc required for targeting PUFc-based effectors to target sites. The sgRNAs used to target *MLH1* promoter regions (A-F), CpGs (lollipops), and TSS (arrow) are depicted above the plot. NS, not significant, *p*>0.05, one-way ANOVA.

Supplementary Figure 4

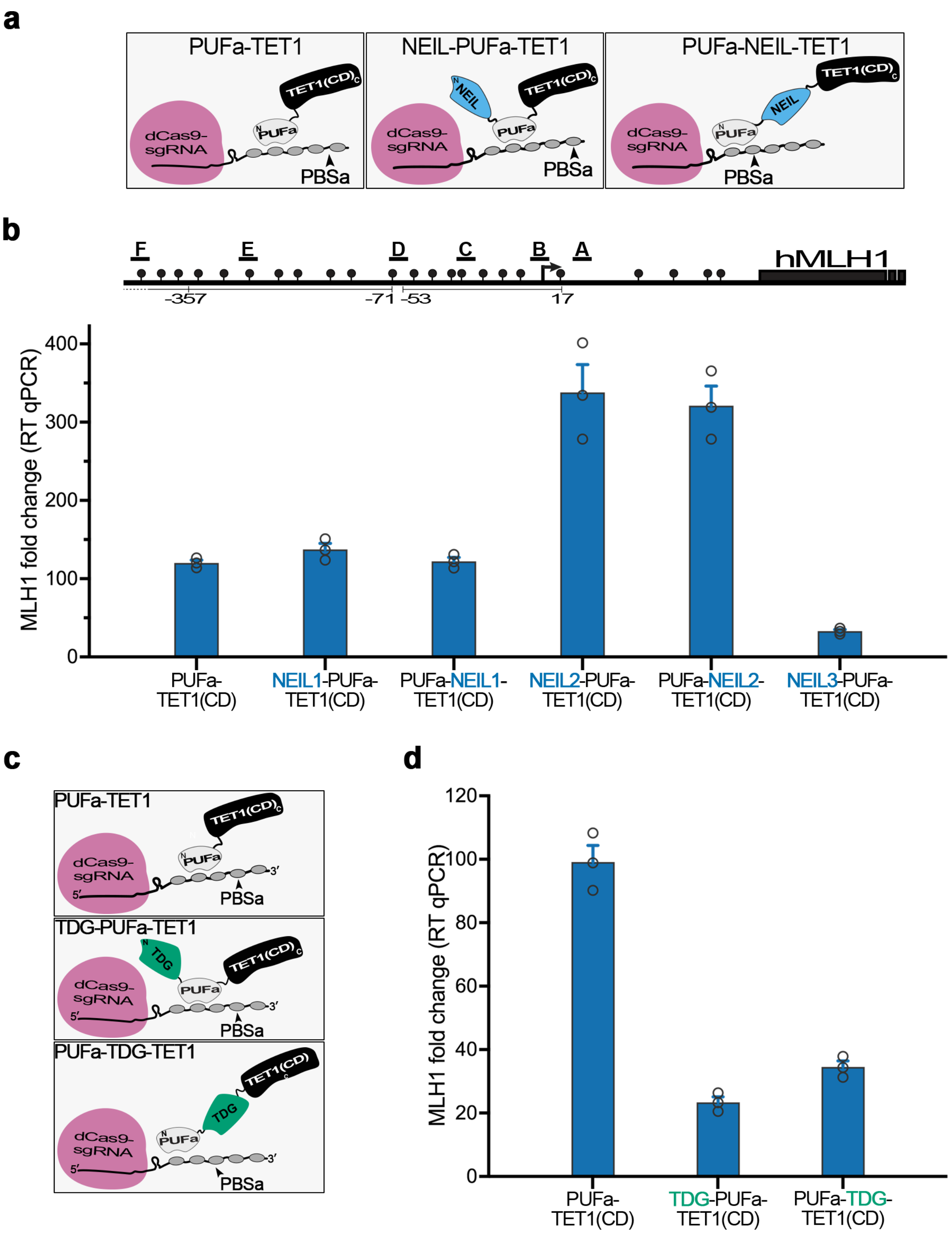

Supplementary Figure 4

Co-targeting TET1 activity and DNA glycosylases to *MLH1* promoter. (a) Schematic representation of *Casilio-ME1* and its derivatives that include NEIL proteins (NEIL1, NEIL2 or

NEIL3 glycosylases) as part of associated TET1 effector protein fusions. TET1(CD) (black), PUFa (light grey), NEIL proteins (blue), amino (N) and carboxyl (C) termini of protein fusion are arbitrarily shown. **(b)** *MLH1* mRNA relative levels (mean fold change ± S.E.M.; n=3) as determined by TaqMan assays in cells transfected in the presence of indicated effectors. A drawing of sgRNAs used to target the *MLH1* promoter regions (A-F), CpGs (lollipops), and TSS (arrow) are shown above the plot. Similar results were obtained when NEIL1 and NEIL3 were used as modular PUFc effectors in the presence of PUFa-TET1(CD) and sgRNAs containing PBSa and PBSc. **(c)** *Casilio-ME1* and its derivatives that include TDG as part of TET1 effector fusions are arbitrarily depicted to show TET1(CD) (black), PUFa (light grey), TDG (green), and amino (N) and carboxyl (C) termini of protein fusions. **(d)** *MLH1* mRNA relative levels (mean fold change ± S.E.M.; n=3) in cells transfected in the presence of indicated effectors and the *MLH1*-sgRNAs shown in (b upper panel)**.** TDG failed to enhance *Casilio-ME* mediated gene activation when linked to TET1 effector although it plays an important role processing 5fC and 5caC. Same results were obtained when TDG was linked to PUFc as modular effectors in the presence of PUFa-TET1(CD) and sgRNAs containing PBSa and PBSc. This failure to enhance TET1-mediated gene activation with TDG could be due to improper folding of the TDG fusion proteins tested. While alternative explanations exist, other factors might be required to permit coupling of TET1 and TDG activities at targeted sites.

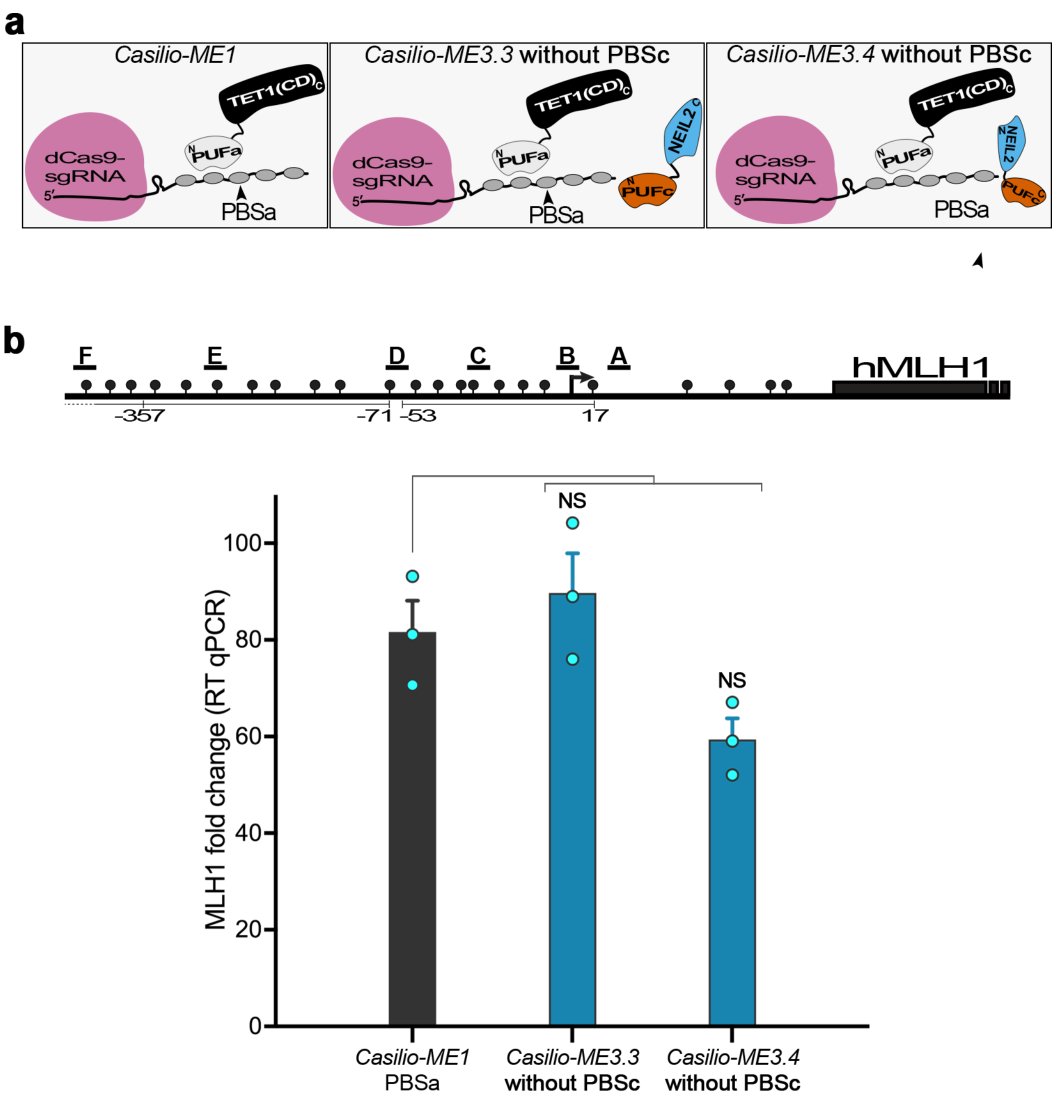

Supplementary Figure 5

Enhanced *MLH1* activation by *Casilio-ME3* platforms requires co-delivery of TET1(CD) and NEIL2 to targeted genomic loci. (a) Representation of *Casilio-ME* platforms showing effector modules of PUFa and PUFc protein fusions utilized to transfect cells in the presence of sgRNAs that contained PBSa but lacked PBSc required for targeting the PUFc-based effectors to genomic sites. TET1(CD) (black), NEIL2 (blue), PUFa (light grey), PUFc (orange), amino (N) and carboxyl (C) termini of protein fusions are arbitrarily shown. **(b)** *MLH1* mRNA relative levels (mean fold change ± S.E.M.; n=3) in cells transfected with components of *Casilio-ME1, Casilio-ME3.3, Casilio-ME3.4* in the presence of sgRNAs that contained PBSa but lacked PBSc required for targeting the PUFc-based effectors to target sites. Drawings of the sgRNAs used to target the *MLH1* promoter regions (A-F), CpGs (lollipops), and TSS (arrow) are shown above the column plot. NS, not significant, $p>0.05$, one-way ANOVA.

Supplementary Figure 6

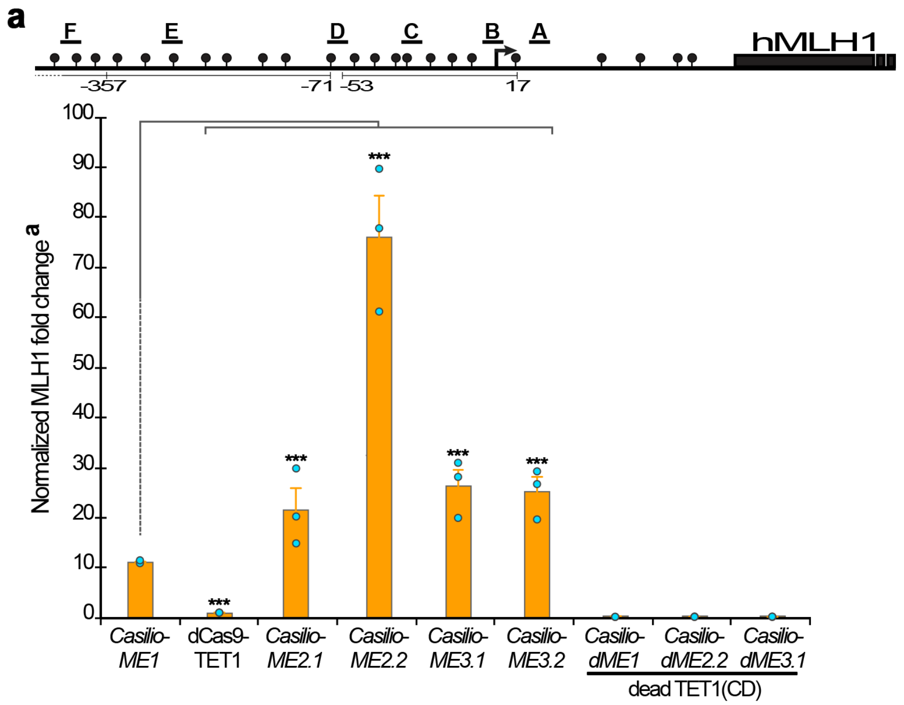

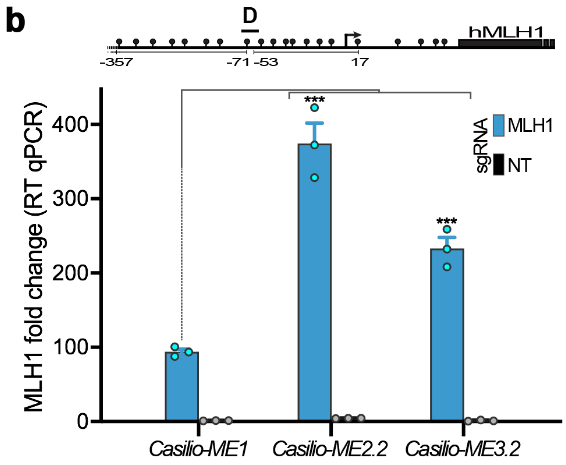

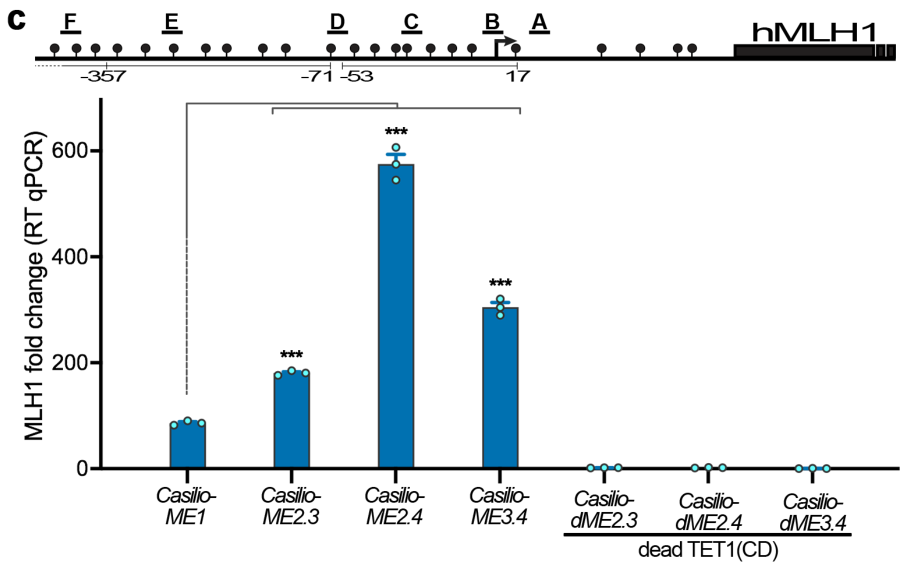

Supplementary Figure 6

Comparison of the enhanced gene activation of *Casilio-ME2* and *Casilio-ME3* platforms. (a) *MLH1* mRNA normalized levels (mean fold change ± S.E.M.; n=3) in cells transfected with components of *Casilio-ME1, Casilio-ME2.1, Casilio-ME2.2, Casilio-ME3.1, Casilio-ME3.2* or dCas9-TET1 systems in the presence of *MLH1*-sgRNAs. In the shown *Casilio-dME* derivatives, TET1(CD) was replaced by dTET1(CD) containing TET1-inactivating mutations. Drawings of the sgRNAs used to target the *MLH1* promoter regions (A-F), CpGs (lollipops), and TSS (arrow) are shown above the column plot. *** $p< 0.0005$, one-way ANOVA.[a/] Fold changes were normalized to those obtained with targeting dCas9-TET1 to *MLH1* promoter which were 19.8 ± 0.7, 27.2 ± 1.8, and 16.7 ± 0.4 (mean fold change ± S.E.M.; n=3) relative to mock transfected cells in the three independent experiments shown, respectively. **(b)** Relative *MLH1* mRNA fold change (mean ± S.E.M.; n=3) in cells transfected with components of *Casilio-ME1, Casilio-ME2.2,* or *Casilio-ME3.2* in the presence of one sgRNA as indicated. *** $p<0.01$, one-way ANOVA. **(c)** Relative levels *MLH1* mRNA (mean ± S.E.M.; n=3) in cells transfected with components of *Casilio-ME1, Casilio-ME2.3, Casilio-ME2.4* or *Casilio-ME3.4* as indicated. *MLH1*-sgRNA used are as shown in (a upper panel) but contained both PBSa and PBSc required for modular targeting of the associated effectors. dead TET1(CD) containing TET1-inactivating mutations replaced TET1(CD) in the shown *Casilio-dME* derivatives. *** $p<0.0001$, one-way ANOVA.

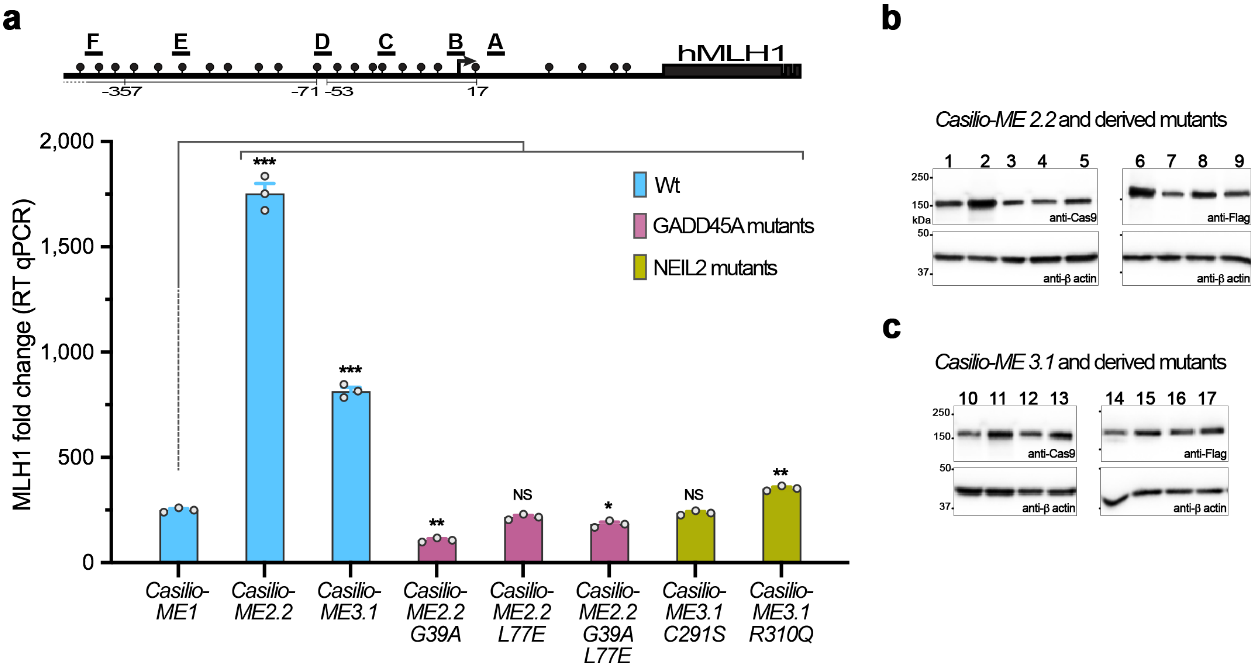

Supplementary Figure 7

Enhanced gene activations with *Casilio-ME2* and *Casilio-ME3* platforms require active GADD45A and NEIL2. (a) Column plot showing *MLH1* mRNA fold change (mean fold change ± S.E.M.; n=3) in cells transfected with components of *Casilio-ME1, Casilio-ME2.2, or Casilio-ME3.1* in the presence of the indicated *MLH1*-sgRNAs. Drawing of promoter regions with the *MLH1*-sgRNAs used (A-F), CpGs (lollipops), and TSS (arrow) is shown above the plot. Wild type (Wt) *Casilio-ME* platforms (blue) and derived *Casilio-ME2.2* and *Casilio-ME3.1* mutants containing the indicated point mutations that alter key functional properties of GADD45A (reddish purple) or inactive NEIL2 (green) are shown. NS, not significant, p>0.05, * p< 0.05, ** p<0.01, *** p<0.0001, one-way ANOVA. **(b, c)** Western blot analysis using anti-Cas9, anti-Flag or anti-β actin antibodies as indicated and protein extracts from cells analyzed for *MLH1* activation in (a). Flag-tagged and dCas9 protein components of wild type and derived mutants of *Casilio-ME* platforms *Casilio-ME2.2* (b) (lanes 1-9) and *Casilio-ME3.1* (c) (lanes 10-15) are shown: Wt (lanes 1, 6, 10 and 14), **d**TET1(CD) (lanes 2, 11 and 15), GADD45A(G39A) (lanes 3 and 7), GADD45A(L77E) (lanes 4 and 8), GADD45A(G39A L77E) (lanes 5 and 9), NEIL2(C291S) (lanes 12 and 16), and NEIL2(R310Q) (lanes 13 and 17).

Supplementary Figure 8

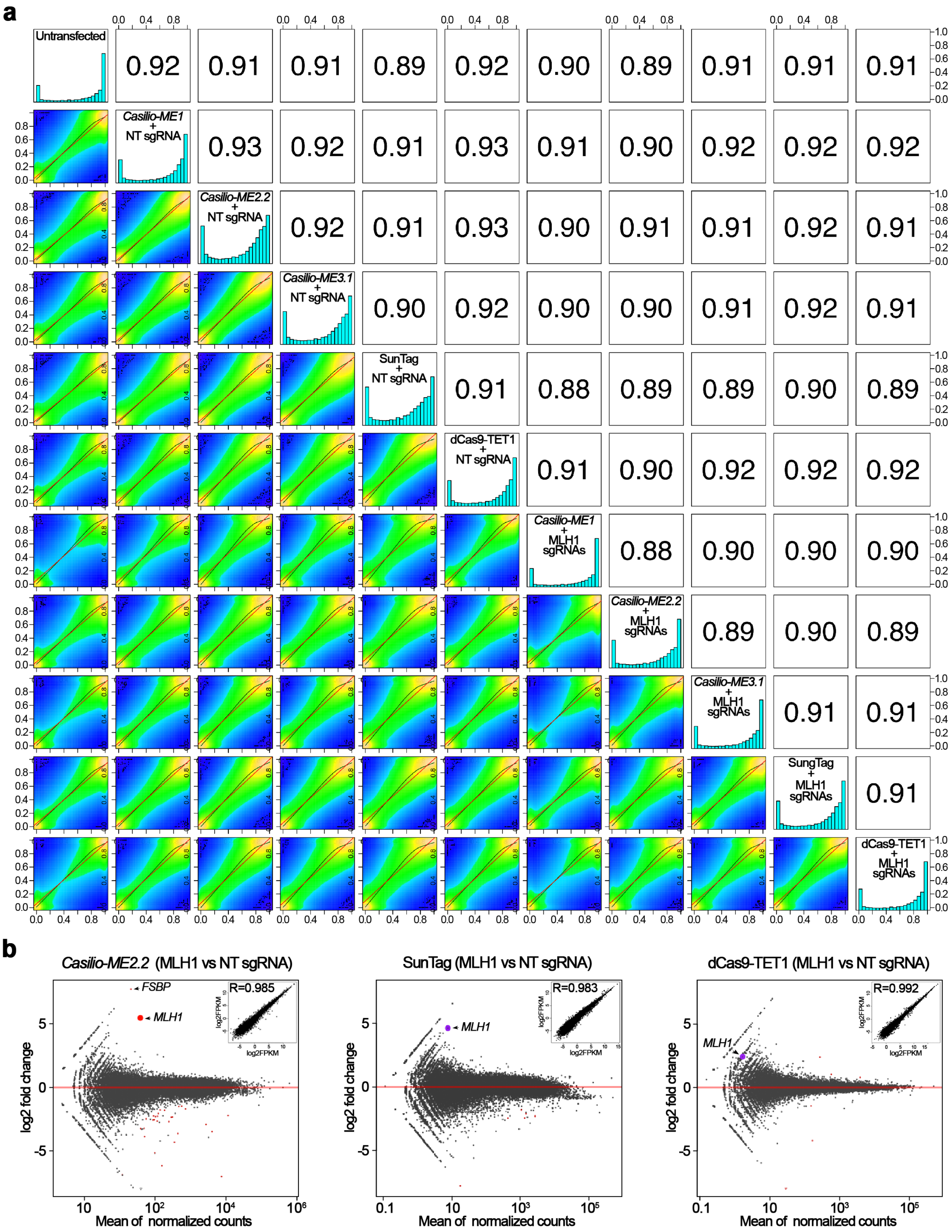

Supplementary Figure 8

Evaluation of potential off-target effects on 5mC demethylation and gene expression. (a) Histograms of CpG genome-wide 5mC methylation frequency and correlations between indicated samples with corresponding Pearson's correlation coefficient are shown. Untransfected cells or cells transfected with indicated 5mC demethylation systems in the presence of targeting *MLH1*-sgRNAs (A-F) or NT-sgRNA are shown. **(b)** RNAseq analysis of two biological replicates of RNA samples obtained from cells transfected with *Casilio-ME2.2*, SunTag or dCas9-TET1 components in the presence of *MLH1*-sgRNAs or a NT-sgRNA are shown. Red dots represent differentially expressed hits deemed significant and purple dots represent non-significant *MLH1* differential expression based on obtained *p* values (see Methods). Pearson's correlation coefficients are shown as insert with corresponding MA-plots.

Supplementary Figure 9

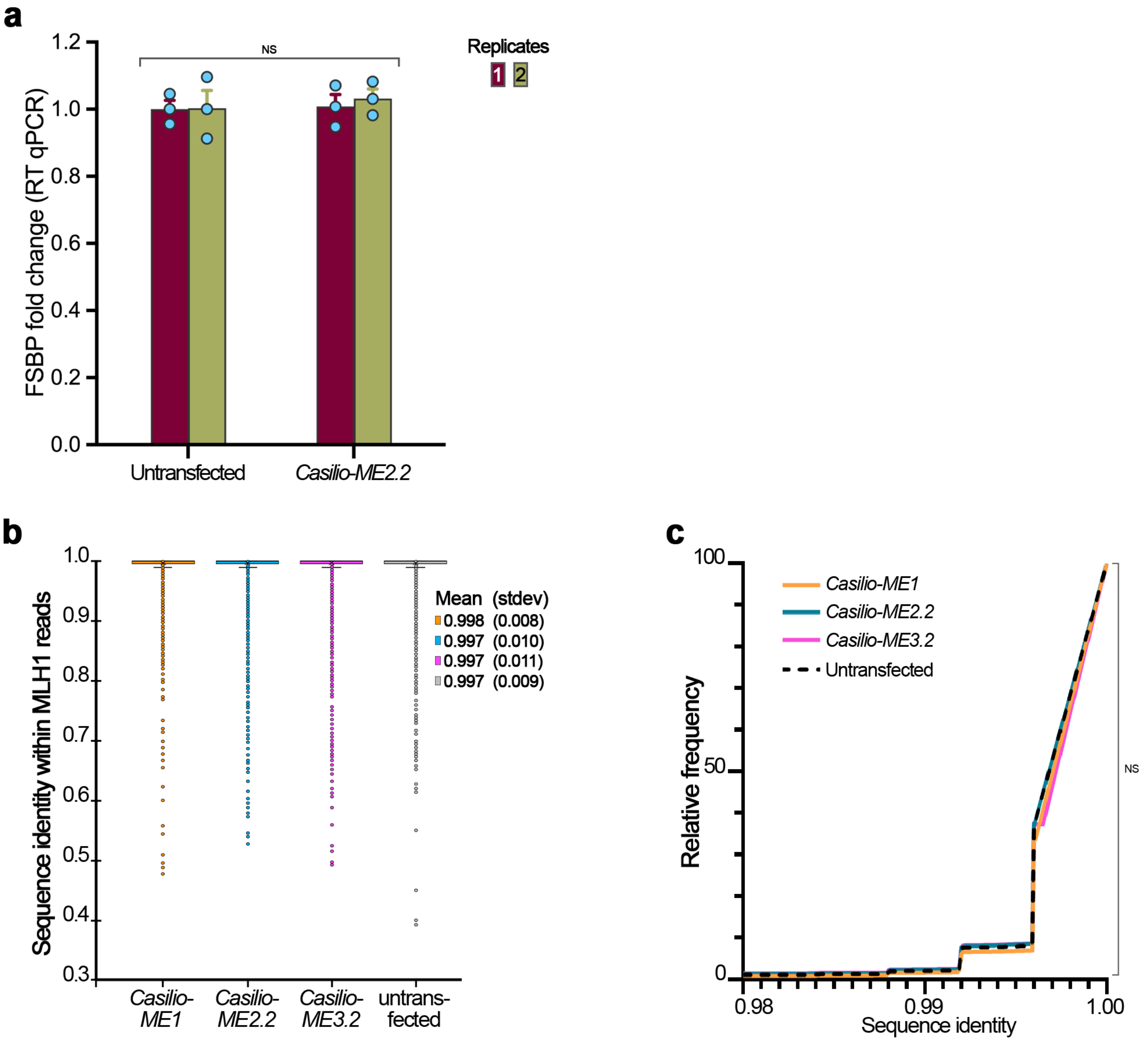

Supplementary Figure 9

Evaluation of potential off-target activation of *FSBP* and mutagenicity of *Casilio-ME* platforms. (a) TaqMan assay showing *FSBP* mRNA relative levels (mean fold change ± S.E.M; n=3) in the two replicates of mRNA samples analyzed by RNAseq shown in (Fig. S8b). NS, not significant, $p>0.05$, two-way ANOVA. **(b**, **c)** Sequence analysis of *MLH1* reads in amplicons obtained from untransfected cells or cells transfected with components of the indicated *Casilio-ME* platforms and the six *MLH1*-sgRNAs. Box plot (b) and cumulative distribution frequency plot (c) of sequence identity distribution in *MLH1* reads. For each box plot the thick line inside the box represents the median value and the surrounding bottom and top lines represent the 25th and 75th percentiles. The whiskers represent min and max values, the x represents the mean value and the dots represent outliers. Statistical significance of differences in sequence identity distribution between samples were tested. NS, not significant, $p>0.5$, one-way ANOVA. $p>0.1$ (untransfected

cells vs *Casilio-ME1*), $p>0.5$ (untransfected cells vs *Casilio-ME2.2*), and $p>0.1$ (untransfected cells vs *Casilio-ME3.2*), Mann-Whitney U test.

Supplementary Figure 10

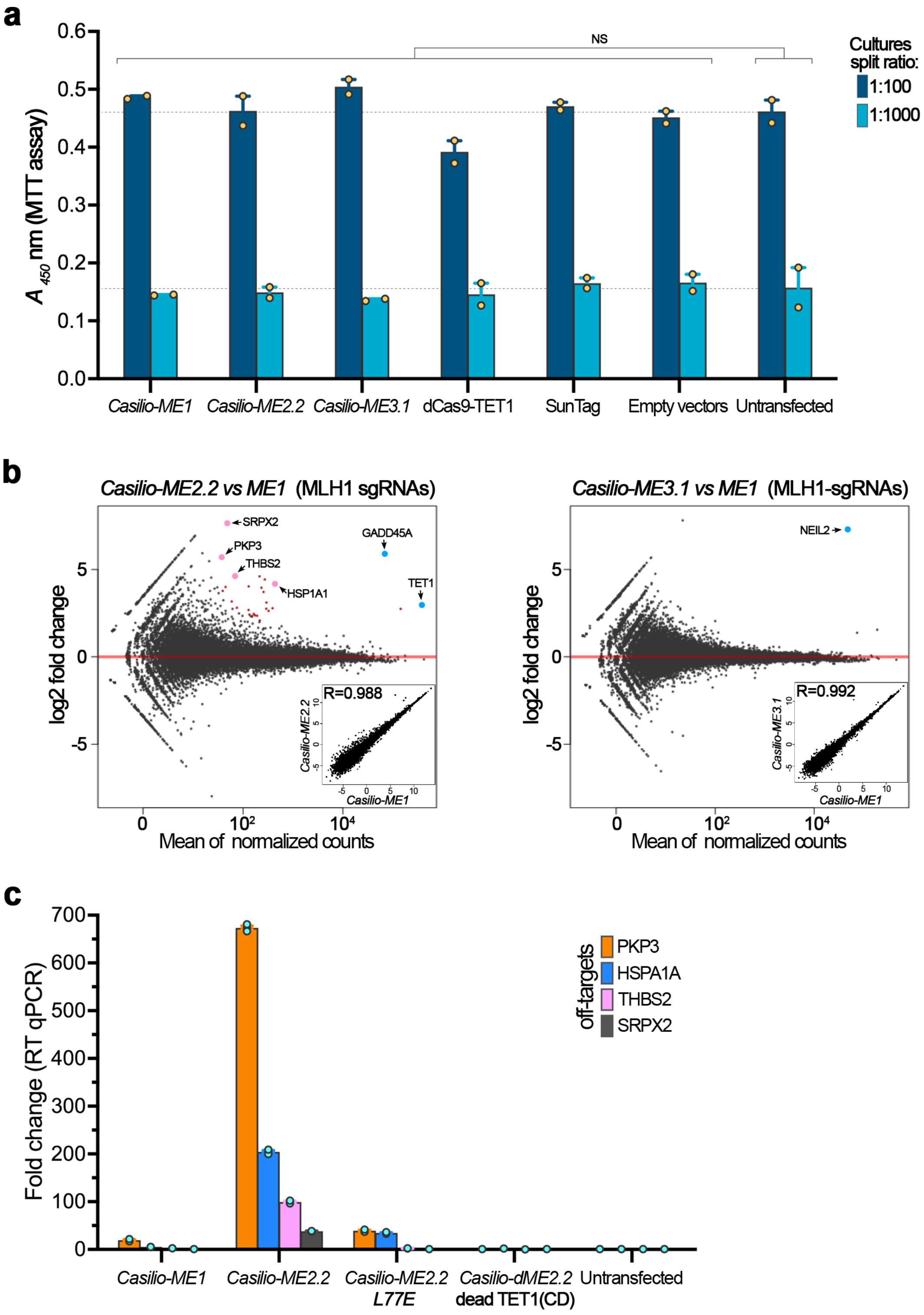

Supplementary Figure 10

Evaluation of the effect of expression of the *Casilio-ME* PUFa-fusion proteins on cell growth and transcriptome. **(a)** MTT cell proliferation assays (Mean A_{540} ± S.E.M; n=2) of untransfected and transfected cells with empty vectors or plasmids of the *Casilio-ME* platforms, SunTag or dCas9-TET1 alternative 5mC demethylation systems. Cultures of untransfected and transfected cells were serially split (1:10, 1:100 and 1:1000) 48h post transfection, and then subjected to MTT assay. Cell cultures that were split at 1:10 ratio all gave saturated absorbance ($A_{450} \approx 4$). NS, not significant, p >0.05, one way ANOVA. **(b)** Evaluation of the effect of GADD45A and NEIL2 expression, as part of PUFa-TET1(CD) fusion-proteins of the *Casilio-ME* platforms, on transcriptomes. RNAseq analysis of two biological replicates of RNA samples obtained from cells transfected with the indicated *Casilio-ME* components in the presence of *MLH1*-sgRNAs are shown. Red dots represent differentially expressed hits deemed significant based on adjusted p values obtained (Methods). Arrows highlight differentially expressed transcripts of *Casilio-ME* components (blue dots) and the off-target genes tested in RT-qPCR shown in (c) (pink dots). Pearson's correlation coefficients are shown as inserts with corresponding log2 FPKM MA-plots. **(c)** mRNA fold changes (mean fold change ± S.E.M.; n=3) of the indicated off-target genes in cells transfected with the six *MLH1* sgRNAs and components of *Casilio-ME1*, *Casilio-ME2.2*, *Casilio-ME2.2* variant comprising GADD45A (L77E) point mutant, or *Casilio-dME2.2* containing catalytically inactive TET1(CD). PKP3, HSP1A1, THBS2 and SRPX2, identified as upregulated off-target genes in RNAseq analysis (b), were chosen to validate RNAseq analysis, and to ask whether expression of GADD45A in the absence TET1 oxidative activity could lead to activated expression of these four genes.

Supplementary Figure 11

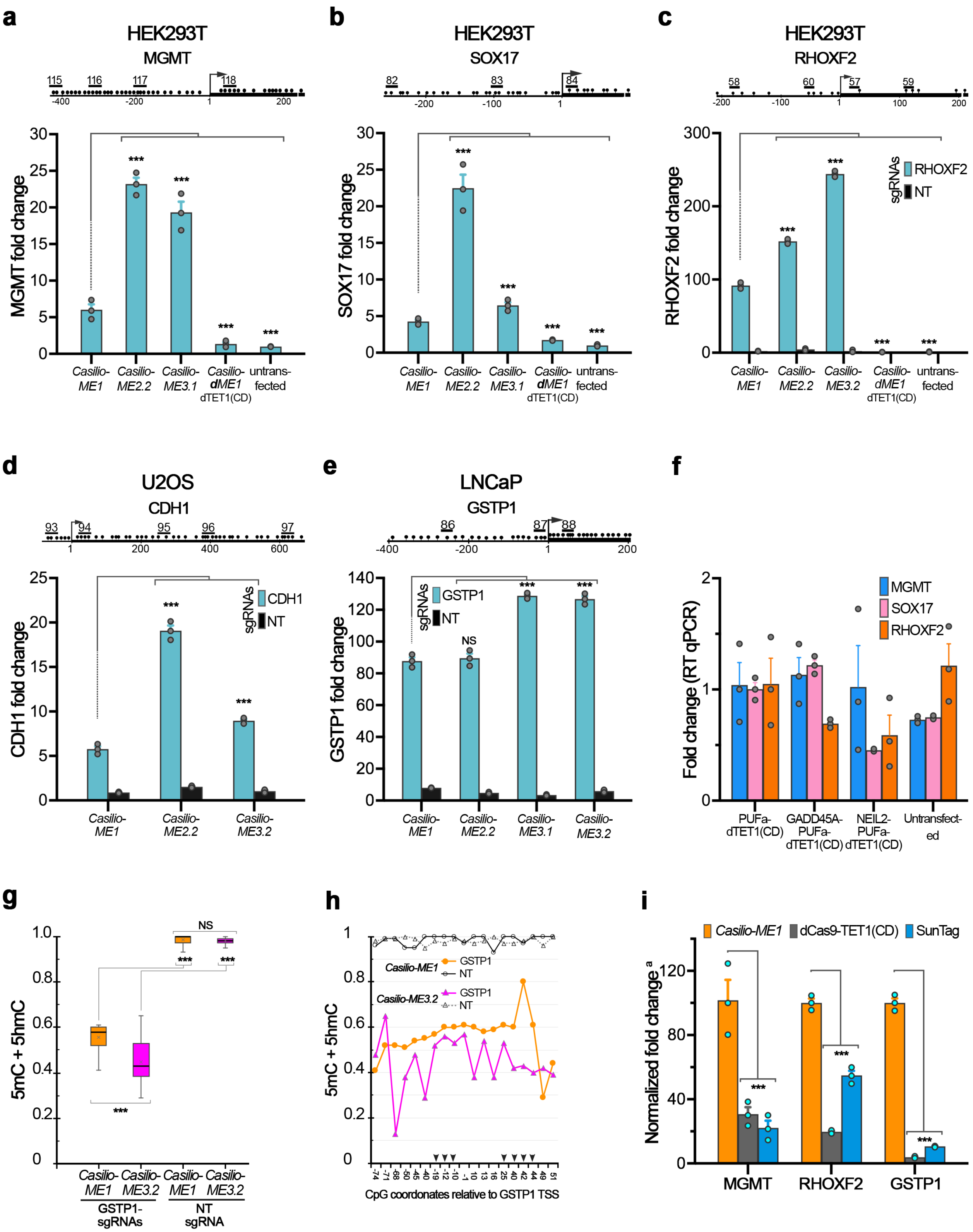

Supplementary Figure 11

***Casilio-ME* platforms enable enhanced activation of methylation-regulated genes. (a-e)** mRNA fold changes (mean fold change ± S.E.M.; n=3) obtained by targeting different genes in different cell types by transfection (HEK293T, U2OS) or nucleofection (LNCaP) of indicated *Casilio-ME* components and sgRNAs are shown. mRNA levels were quantitated in cells collected 3 days after transfection. When indicated TET1(CD) was replaced with catalytically dead TET(CD) to show the role of TET1 oxidative activity in the obtained gene activations. The targeted promoter regions with associated CGI depicting TSS (arrow), CpG (lollipops) and location of sgRNAs used (lines under numbers) are shown above corresponding plot. NS, not significant, p>0.05, *** p< 0.005, one-way ANOVA. **(f)** mRNA fold changes (mean fold change ± S.E.M.; n=3) in cells transfected with the indicated PUFa-fusion proteins containing catalytically inactive TET1(CD) in the absence of targeting sgRNAs. **(g, h)** Efficiency of 5mC demethylation induced by targeting the indicated *Casilio-ME* components to *GSPT1* CGI of LNCaP cells in the presence of GSTP1-targeting sgRNAs or NT-sgRNA. Methylation frequencies were determined by high throughput amplicon BSeq of genomic DNA derived from cells analyzed in panel (e). CpGs overlapping sgRNA target sequences are shown (arrow heads). For each box plot the thick line inside the box represents the median value and the surrounding bottom and top lines represent the 25th and 75th percentiles. The whiskers represent min and max values, the x represents the mean value. NS, not significant, p>0.05, *** p< 0.005, one-way ANOVA. **(i)** Normalized mRNA fold changes of the indicated methylation-silenced genes (mean fold change ± S.E.M.; n=3) in cells transfected by *Casilio-ME1*, dCas9-TET1(CD) or SunTag components in the presence of the respective targeting sgRNA as shown in panels a, c and e. *** p< 0.001, two-way ANOVA.

[a/] Obtained fold changes with *Casilio-ME1* targeting *MGMT*, *RHOXF2* or *GSTP1* CGIs were 5.92 ± 0.76, 89.43 ± 2.35 and 79.66 ± 2.29, respectively.

Supplementary Figure 12

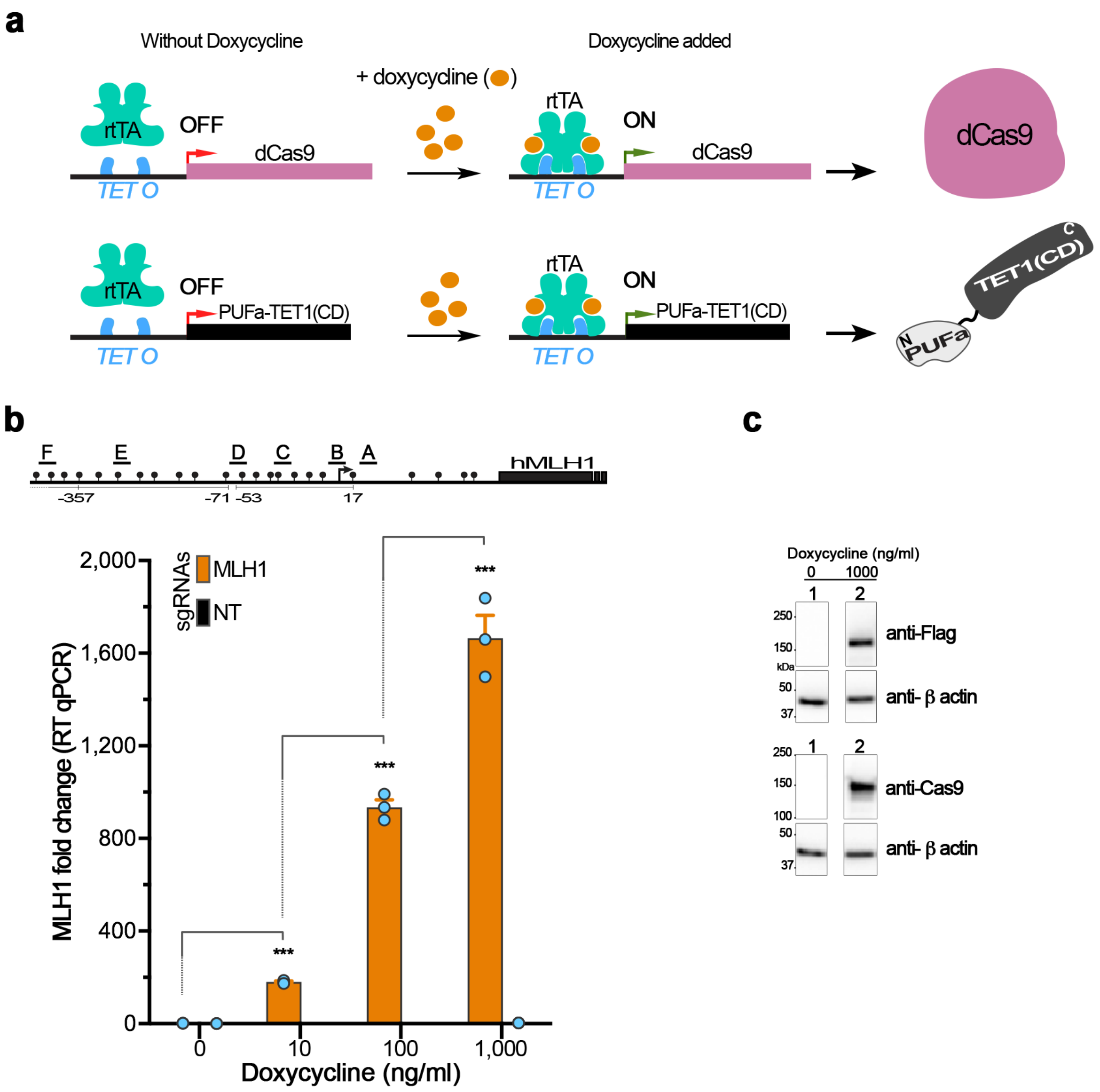

Supplementary Figure 12

Tunable activation of methylation-silenced genes. (a) Depiction of expression inducible *Casilio-ME1* components using Tet-ON system. The reverse tetracycline-controlled transcriptional activator (r-tTA) does not bind *Tet* operator (TET O) sequences in the absence of doxycycline. Doxycycline-bound rtTA undergoes conformational changes allowing its binding to TET O sequences and inducing subsequent transcriptional activation of associated genes. **(b)** Range of *MLH1* activation (mean mRNA fold change ± S.E.M.; n=3) obtained with *DIP_Casilio-ME1* platform when media were supplemented with a range of Dox concentration. PiggyBac vectors hosting cassette enabling a Dox-inducible expression of dCas9 or PUFa-TET1(CD) effector were concomitantly delivered to cells via PiggyBac transposase system[1 2], and doubly selected cells were then transiently transfected with *MLH1*-sgRNAs. Depiction of *MLH1* promoter showing sgRNAs used is shown above the plot. *** $p<0.0001$, one-way ANOVA. **(c)** Western blot analysis using anti-Flag, anti-Cas9, anti-β actin monoclonal antibodies and protein extracts form

DIP_*Casilio-ME1* cells transfected with *MLH1*-sgRNAs without (lanes 1) or with 1µg/ml (lane 2) supplemented doxycycline is shown.

Supplementary Table 1- Plasmid list

Plasmid ID	Description	Addgene ID
pAC1371	pX-sgRNA-5xPBSa- Cloning vector to express sgRNA-5xPBSa	71888
pAT243	pX-sgRNA-5xPBSa- Cloning vector to express sgRNA-5xPBSa_5xPBSc	Pending
pAT888	CMV/CAG_PUFa-hTET1(CD)	Pending
pAC1445	pmax_dCas9	73169
pAT890	CMV/CAG_dCas9-hTET1(CD)	Pending
pCAG-dCas9-5xPlat2AflD	For dCas9-(SunTag)x5 array expression	82560
pCAG-scFvGCN4sfGFPTET1CD	For antibody-sfGFP-hTET1(CD) expression	82561
pcDNA3.1-MS2-Tet1-CD	For MS2 coat protein-mTET1(CD) expression	83341
pdCas9-Tet1-CD	For dCas9-TET1(CD) and sgRNA expression	83340
pAT801	CMV/CAG_TALE_A-hTET1(CD)	Pending
pAT812	CMV/CAG_TALE_B-hTET1(CD)	Pending
pAT806	CMV/CAG_TALE_D-hTET1(CD)	Pending
pAT817	CMV/CAG_TALE_F-hTET1(CD)	Pending
pAT341	CMV/CAG_PUFa-TET1CD (H1671Y D1673A) (dead TET1)	Pending
pAT360	CMV/CAG_PUFa-hGADD45A-hTET1(CD)	Pending
pAT635	CMV/CAG_hGADD45A-PUFa-hTET1(CD)	Pending
pAT355	CMV/CAG_PUFc-hGADD45A	Pending
pAT356	CMV/CAG_hGADD45A-PUFc	Pending
pAT892	CMV/CAG_PUFa-p65HSF1	Pending
pAT608	CMV/CAG_Flag-hNEIL2-PUFa-hTET1(CD)	Pending
pAT611	CMV/CAG_PUFa-hNEIL2-hTET1(CD)	Pending
pAT595	CMV/CAG_PUFc-hNEIL2	Pending
pAT596	CMV/CAG_hNEIL2-PUFc	Pending
pAT607	CMV/CAG_hNEIL1-PUFa-hTET1(CD)	N/A
pAT610	CMV/CAG_PUFa-hNEIL1-hTET1(CD)	N/A
pAT609	CMV/CAG_hNEIL3-PUFa-hTET1(CD)	N/A
pAT359	CMV/CAG_PUFa-hTDG-hTET1(CD)	N/A

pAT335	CMV/CAG_hTDG-PUFa-hTET1(CD)	N/A
pAT699	CMV/CAG_Flag-hGADD45A-PUFa-hTET1(CD)	Pending
pAT976	CMV/CAG_Flag-hGADD45A-PUFa-dTET1CD	Pending
pAT971	CMV/CAG_Flag-hGADD45A (G39A)-PUFa-hTET1(CD)	Pending
pAT972	CMV/CAG_Flag-hGADD45A (L77E)-PUFa-hTET1(CD)	Pending
pAT973	CMV/CAG_FlaghGADD45A (G39A, L77E)-PUFa-hTET1(CD)	Pending
pAT977	CMV/CAG_Flag-hNEIL2-PUFa-dTET1(CD)	Pending
pAT1059	CMV/CAG_Flag-hNEIL2 (C291S)-PUFa-hTET1(CD)	Pending
pAT1060	CMV/CAG_Flag-hNEIL2 (R310Q)-PUFa-hTET1(CD)	Pending
pAT1089	PB-EF1a-Blast_2A_rtTA3-SV40pA_pCW-dCas9	Pending
pAT1090	PB-EF1a-Hygro_2A_rtTA3-SV40pA_pCW-Flag-PUFa-hTET1(CD)	Pending

Supplementary Table 2- List of sgRNA spacer sequences.

sgRNAs	spacer sequences	References
MLH1-A	gACAGAGTTGAGAAATTTGAC	This work
MLH1-B	GGCAGTAGCCGCTTCAGGGA	This work
MLH1-C	GCGCAAGCGCATATCCTTCT	This work
MLH1-D	gAAACGAACCAATAGGAAGAG	This work
MLH1-E	GCGCCAGATCACCTCAGCAG	This work
MLH1-F	gCTGACGCAGACGCTCCACCA	This work
Non targeting	gTTCTCTTGCTGAAAGCTCGA	[3]
RHOXF2-57	gCCCGCTATTTGCTGTGGGTT	[4]
RHOXF2-58	gACTCACGCATGCCTGTCTAC	This work
RHOXF2-59	gTAGCACTGCCTAGGAGAGCG	This work
RHOXF2-60	gAACGCGTGCTCTCCCTCATC	This work
SOX17-82	GTACAATCAGCCCTCCCAGA	This work
SOX17-83	GTGGGACTCGGACCACGGCC	This work
SOX17-84	gTCTGTGCAGAAAAGGCCCCG	This work
GSTP1-85	GAAGCGGGTGTGCAAGCTCC	This work
GSTP1-86	GTTTACTCCCTAGGCCCCGC	This work
GSTP1-87	gTATAAGGCTCGGAGGCCGCG	This work
GSTP1-88	gTCGCCACCAGTGAGTACGCG	This work
CDH1-93	GCGTCTATGCGAGGCCGGGT	This work
CDH1-94	GTACGGGGGGCGGTGCCTCCG	This work
CDH1-95	gCCGGATCCCCTGACTTGCGA	This work
CDH1-96	GCCTGGAAGCCTCGCGCGCTC	This work
CDH1-97	gAGTCGTGGGGACGATCTTCG	This work
MGMT-114	GGACCGGGATTCTCACTAAG	This work
MGMT-115	GCAGGTCGCTTGCACGCCCG	This work
MGMT-116	GCCCGGCTTGTACCGGCCGA	This work
MGMT-117	GCACAGGGCATGCGCCGACC	This work

Supplementary Table 3- Sequence of the sgRNA scaffold with 5xPBSa and 5xPBSc

Description	DNA Sequence
U6 promoter-sgRNA-5x**PBSa-**PBSc For expression of modified sgRNAs comprising spacer sequence (*N*s) inserted at the 5'end, and a set of five binding sites for PUFa (PBSa, bold face) or PUFc (PBSc, underlined) added to the 3'region of the sgRNA.	gagggcctatttcccatgattccttcatatttgcatatacgatacaaggctgttagagagataattggaattaatttgactgtaaacacaaagatattagtacaaaatacgtgacgtagaaagtaataatttcttgggtagtttgcagttttaaaattatgttttaaaatggactatcatatgcttaccgtaacttgaaagtatttcgatttcttggctttatatatcttGTGGAAAGGACGAAACACC*gNNNNNNNNNNNNNNNNNNN*GTTTaagagctaTGCTGGAAACAGCAtagcaagttTaaataaggctagtccgttatcaacttgaaaaagtggcaccgagtcggtgcCAATTGgtctccagat**TGTATGTA**gcc**TGTATGTA**gcc**TGTATGTA**gcc**TGTATGTA**gcc**TGTATGTA**aGATCCAATTGggtctccagatTTGATGTAgccTTGATGTAgccTTGATGTAgccTTGATGTAgccTTGATGTAagatCTTTTTTTgttttagagctagaaatagcaagttaaaataaggctagtccgtagcgcgtgcgccaattctgcagacaaatggc

U6-sgRNA containing 5 PBSa, Addgene ID #71888 was as previously reported [3].

Supplementary Table 4- List of bisulfite sequencing PCR primers

Primers	Primer sequences	Targeted promoters
AT442-F	gatccccgggtaccgagctcgaattAAGGTTAAGAGGYGGTAGAGTT	*MLH1* distal (cloning)
AT443-R	cgttgtaaaacgacggccagtgaattTTAACCCTACTCTTATAACCTCCC	
AT444-F	gatccccgggtaccgagctcgaattGGGAGGTTATAAGAGTAGGGTTAA	*MLH1* intermediate (cloning)
AT445-R	cgttgtaaaacgacggccagtgaattCATCCAACCCCACCCTTCAA	
AT446-F	gatccccgggtaccgagctcgaattTTGAAGGGTGGGGTTGGATG	*MLH1* proximal (cloning)
AT447-R	cgttgtaaaacgacggccagtgaattTTATAAACATACRCTATACATACCTCTACC	
AT531-F	AAGGTTAAGAGGYGGTAGAGTT	*MLH1* distal
AT532-R	TTAACCCTACTCTTATAACCTCCC	
AT533-F	GGGAGGTTATAAGAGTAGGGTTAA	*MLH1* intermediate
AT534-R	CATCCAACCCCACCCTTCAA	
AT535-F	TTGAAGGGTGGGGTTGGATG	*MLH1* proximal
AT536-R	TTATAAACATACRCTATACATACCTCTACC	
AT541-F	GGTGTGTAAGTTTYGGGAT	*GSTP1*
AT542-R	AAAAACCCTACCRAAAACCC	

Supplementary Table 5- List of relevant protein sequences

Name: **PUFa-TET1(CD)**
**Keys:** NLS, PUFa, **TET1(1418-2136)**
MIDGGGGSDPKKKRKVDPKKKRKVDPKKKRKVGSTGSRNDGGGGSGGGGSGGGGSGRAGILPPKKK<br>RKVSRGRSRLLEDFRNNRYPNLQLREIAGHIMEFSQDQHGSRFIQLKLERATPAERQLVFNEILQAAYQL<br>MVDVFGNYVIQKFFEFGSLEQKLALAERIRGHVLSLALQMYGSRVIEKALEFIPSDQQNEMVRELDGHVL<br>KCVKDQNGNHVVQKCIECVQPQSLQFIIDAFKGQVFALSTHPYGCRVIQRILEHCLPDQTLPILEELHQHT<br>EQLVQDQYGNYVIQHVLEHGRPEDKSKIVAEIRGNVLVLSQHKFASNVVEKCVTHASRTERAVLIDEVCT<br>MNDGPHSALYTMMKDQYANYVVQKMIDVAEPGQRKIVMHKIRPHIATLRKYTYGKHILAKLEKYYMKNGV<br>DLGDPKKKRKVDPKKKRKVGGRGGGGSGGGGSGGGGSGPA**ELPTCSCLDRVIQKDKGPYYTHLGAG**<br>**PSVAAVREIMENRYGQKGNAIRIEIVVYTGKEGKSSHGCPIAKWVLRRSSDEEKVLCLVRQRTGHHCPT**<br>**AVMVVLIMVWDGIPLPMADRLYTELTENLKSYNGHPTDRRCTLNENRTCTCQGIDPETCGASFSFGCS**<br>**WSMYFNGCKFGRSPSPRRFRIDPSSPLHEKNLEDNLQSLATRLAPIYKQYAPVAYQNQVEYENVAREC**<br>**RLGSKEGRPFSGVTACLDFCAHPHRDIHNMNNGSTVVCTLTREDNRSLGVIPQDEQLHVLPLYKLSDT**<br>**DEFGSKEGMEAKIKSGAIEVLAPRRKKRTCFTQPVPRSGKKRAAMMTEVLAHKIRAVEKKPIPRIKRKN**<br>**NSTTTNNSKPSSLPTLGSNTETVQPEVKSETEPHFILKSSDNTKTYSLMPSAPHPVKEASPGFSWSPKT**<br>**ASATPAPLKNDATASCGFSERSSTPHCTMPSGRLSGANAAAADGPGISQLGEVAPLPTLSAPVMEPLI**<br>**NSEPSTGVTEPLTPHQPNHQPSFLTSPQDLASSPMEEDEQHSEADEPPSDEPLSDDPLSPAEEKLPHI**<br>**DEYWSDSEHIFLDANIGGVAIAPAHGSVLIECARRELHATTPVEHPNRNHPTRLSLVFYQHKNLNKPQH**<br>**GFELNKIKFEAKEAKNKKMKASEQKDQAANEGPEQSSEVNELNQIPSHKALTLTHDNVVTVSPYALTH**<br>**VAGPYNHWV**ID
Name: **dCas9**
**Keys:** NLS, *Sp* dCas9, HA tag
MIDGGGGSGGGGSGGGGSMYPYDVPDYASPKKKRKVEASDKKYSIGLAIGTNSVGWAVITDEYKVPSK<br>KFKVLGNTDRHSIKKNLIGALLFDSGETAEATRLKRTARRRYTRRKNRICYLQEIFSNEMAKVDDSFFHRL<br>EESFLVEEDKKHERHPIFGNIVDEVAYHEKYPTIYHLRKKLVDSTDKADLRLIYLALAHMIKFRGHFLIEGDL<br>NPDNSDVDKLFIQLVQTYNQLFEENPINASGVDAKAILSARLSKSRRLENLIAQLPGEKKNGLFGNLIALSL<br>GLTPNFKSNFDLAEDAKLQLSKDTYDDDLDNLLAQIGDQYADLFLAAKNLSDAILLSDILRVNTEITKAPLSA<br>SMIKRYDEHHQDLTLLKALVRQQLPEKYKEIFFDQSKNGYAGYIDGGASQEEFYKFIKPILEKMDGTEELL<br>VKLNREDLLRKQRTFDNGSIPHQIHLGELHAILRRQEDFYPFLKDNREKIEKILTFRIPYYVGPLARGNSRF<br>AWMTRKSEETITPWNFEEVVDKGASAQSFIERMTNFDKNLPNEKVLPKHSLLYEYFTVYNELTKVKYVTE<br>GMRKPAFLSGEQKKAIVDLLFKTNRKVTVKQLKEDYFKKIECFDSVEISGVEDRFNASLGTYHDLLKIIKDK<br>DFLDNEENEDILEDIVLTLTLFEDREMIEERLKTYAHLFDDKVMKQLKRRRYTGWGRLSRKLINGIRDKQS<br>GKTILDFLKSDGFANRNFMQLIHDDSLTFKEDIQKAQVSGQGDSLHEHIANLAGSPAIKKGILQTVKVVDEL<br>VKVMGRHKPENIVIEMARENQTTQKGQKNSRERMKRIEEGIKELGSQILKEHPVENTQLQNEKLYLYYLQ<br>NGRDMYVDQELDINRLSDYDVDAIVPQSFLKDDSIDNKVLTRSDKNRGKSDNVPSEEVVKKMKNYWRQL<br>LNAKLITQRKFDNLTKAERGGLSELDKAGFIKRQLVETRQITKHVAQILDSRMNTKYDENDKLIREVKVITLK<br>SKLVSDFRKDFQFYKVREINNYHHAHDAYLNAVVGTALIKKYPKLESEFVYGDYKVYDVRKMIAKSEQEIG<br>KATAKYFFYSNIMNFFKTEITLANGEIRKRPLIETNGETGEIVWDKGRDFATVRKVLSMPQVNIVKKTEVQT<br>GGFSKESILPKRNSDKLIARKKDWDPKKYGGFDSPTVAYSVLVVAKVEKGKSKKLKSVKELLGITIMERSS<br>FEKNPIDFLEAKGYKEVKKDLIIKLPKYSLFELENGRKRMLASAGELQKGNELALPSKYVNFLYLASHYEKL<br>KGSPEDNEQKQLFVEQHKHYLDEIIEQISEFSKRVILADANLDKVLSAYNKHRDKPIREQAENIIHLFTLTNL<br>GAPAAFKYFDTTIDRKRYTSTKEVLDATLIHQSITGLYETRIDLSQLGGDSPKKKRKVEASGGGGSGGGG<br>SGGGGSGPA
**Name: dCas9-TET1(CD)**
**Keys:** NLS, *Sp* dCas9, **TET1(1418-2136)**, HA tag
MIDGGGGSGGGGSGGGGSMYPYDVPDYASPKKKRKVEASDKKYSIGLAIGTNSVGWAVITDEYKVPSK<br>KFKVLGNTDRHSIKKNLIGALLFDSGETAEATRLKRTARRRYTRRKNRICYLQEIFSNEMAKVDDSFFHRL<br>EESFLVEEDKKHERHPIFGNIVDEVAYHEKYPTIYHLRKKLVDSTDKADLRLIYLALAHMIKFRGHFLIEGDL

NPDNSDVDKLFIQLVQTYNQLFEENPINASGVDAKAILSARLSKSRRLENLIAQLPGEKKNGLFGNLIALSL
GLTPNFKSNFDLAEDAKLQLSKDTYDDDLDNLLAQIGDQYADLFLAAKNLSDAILLSDILRVNTEITKAPLSA
SMIKRYDEHHQDLTLLKALVRQQLPEKYKEIFFDQSKNGYAGYIDGGASQEEFYKFIKPILEKMDGTEELL
VKLNREDLLRKQRTFDNGSIPHQIHLGELHAILRRQEDFYPFLKDNREKIEKILTFRIPYYVGPLARGNSRF
AWMTRKSEETITPWNFEEVVDKGASAQSFIERMTNFDKNLPNEKVLPKHSLLYEYFTVYNELTKVKYVTE
GMRKPAFLSGEQKKAIVDLLFKTNRKVTVKQLKEDYFKKIECFDSVEISGVEDRFNASLGTYHDLLKIIKDK
DFLDNEENEDILEDIVLTLTLFEDREMIEERLKTYAHLFDDKVMKQLKRRRYTGWGRLSRKLINGIRDKQS
GKTILDFLKSDGFANRNFMQLIHDDSLTFKEDIQKAQVSGQGDSLHEHIANLAGSPAIKKGILQTVKVVDEL
VKVMGRHKPENIVIEMARENQTTQKGQKNSRERMKRIEEGIKELGSQILKEHPVENTQLQNEKLYLYYLQ
NGRDMYVDQELDINRLSDYDVDAIVPQSFLKDDSIDNKVLTRSDKNRGKSDNVPSEEVVKKMKNYWRQL
LNAKLITQRKFDNLTKAERGGLSELDKAGFIKRQLVETRQITKHVAQILDSRMNTKYDENDKLIREVKVITLK
SKLVSDFRKDFQFYKVREINNYHHAHDAYLNAVVGTALIKKYPKLESEFVYGDYKVYDVRKMIAKSEQEIG
KATAKYFFYSNIMNFFKTEITLANGEIRKRPLIETNGETGEIVWDKGRDFATVRKVLSMPQVNIVKKTEVQT
GGFSKESILPKRNSDKLIARKKDWDPKKYGGFDSPTVAYSVLVVAKVEKGKSKKLKSVKELLGITIMERSS
FEKNPIDFLEAKGYKEVKKDLIIKLPKYSLFELENGRKRMLASAGELQKGNELALPSKYVNFLYLASHYEKL
KGSPEDNEQKQLFVEQHKHYLDEIIEQISEFSKRVILADANLDKVLSAYNKHRDKPIREQAENIIHLFTLTNL
GAPAAFKYFDTTIDRKRYTSTKEVLDATLIHQSITGLYETRIDLSQLGGDSPKKKRKVEASGGGGSGGGG
SGGGGSGPA**ELPTCSCLDRVIQKDKGPYYTHLGAGPSVAAVREIMENRYGQKGNAIRIEIVVYTGKEGK**
SSHGCPIAKWVLRRSSDEEKVLCLVRQRTGHHCPTAVMVVLIMVWDGIPLPMADRLYTELTENLKSYN
GHPTDRRCTLNENRTCTCQGIDPETCGASFSFGCSWSMYFNGCKFGRSPSPRRFRIDPSSPLHEKNLE
DNLQSLATRLAPIYKQYAPVAYQNQVEYENVARECRLGSKEGRPFSGVTACLDFCAHPHRDIHNMNNG
STVVCTLTREDNRSLGVIPQDEQLHVLPLYKLSDTDEFGSKEGMEAKIKSGAIEVLAPRRKKRTCFTQP
VPRSGKKRAAMMTEVLAHKIRAVEKKPIPRIKRKNNSTTTNNSKPSSLPTLGSNTETVQPEVKSETEPH
FILKSSDNTKTYSLMPSAPHPVKEASPGFSWSPKTASATPAPLKNDATASCGFSERSSTPHCTMPSGR
LSGANAAAADGPGISQLGEVAPLPTLSAPVMEPLINSEPSTGVTEPLTPHQPNHQPSFLTSPQDLASSP
MEEDEQHSEADEPPSDEPLSDDPLSPAEEKLPHIDEYWSDSEHIFLDANIGGVAIAPAHGSVLIECARRE
LHATTPVEHPNRNHPTRLSLVFYQHKNLNKPQHGFELNKIKFEAKEAKNKKMKASEQKDQAANEGPE
QSSEVNELNQIPSHKALTLTHDNVVTVSPYALTHVAGPYNHWVID

Name: **TALE_A-TET1(CD)**

Keys: NLS, TALE_A **TET1(1418-2136)**, His tag

MHHHHHHIDGGGGSDPKKKRKVDPKKKRKVDPKKKRKVGSTGSRNDGGGGSGGGGSGGGGSGRAV
DLRTLGYSQQQQEKIKPKVRSTVAQHHEALVGHGFTHAHIVALSQHPAALGTVAVTYQHIITALPEATHED
IVGVGKQWSGARALEALLTDAGELRGPPLQLDTGQLVKIAKRGGVTAMEAVHASRNALTGAPLNLTPDQ
VVAIAS**NN**GGKQALETVQRLLPVLCQDHGLTPDQVVAIAS**NI**GGKQALETVQRLLPVLCQDHGLTPDQVV
AIAS**NN**GGKQALETVQRLLPVLCQDHGLTPDQVVAIAS**NI**GGKQALETVQRLLPVLCQDHGLTPDQVVAIA
S**NI**GGKQALETVQRLLPVLCQDHGLTPDQVVAIAS**NI**GGKQALETVQRLLPVLCQDHGLTPDQVVAIAS**N**
GGGKQALETVQRLLPVLCQDHGLTPDQVVAIAS**NG**GGKQALETVQRLLPVLCQDHGLTPDQVVAIAS**NG**
GGKQALETVQRLLPVLCQDHGLTPDQVVAIAS**NN**GGKQALETVQRLLPVLCQDHGLTPDQVVAIAS**NI**GG
KQALETVQRLLPVLCQDHGLTPDQVVAIAS**HD**GGKQALETVQRLLPVLCQDHGLTPDQVVAIAS**NG**GGK
QALETVQRLLPVLCQDHGLTPDQVVAIAS**NN**GGKQALETVQRLLPVLCQDHGLTPDQVVAIAS**NN**GGKQ
ALETVQRLLPVLCQDHGLTPDQVVAIAS**HD**GGKQALETVQRLLPVLCQDHGLTPDQVVAIAS**NI**GGKQAL
ETVQRLLPVLCQDHGLTPDQVVAIAS**NG**GGKQALESIVAQLSRPDPALAALTNDHLVALACLGGRPAMDA
VKKGLPHAPELIRRVNRRIGERTSHRVARDPKKKRKVDPKKKRKVGGRGGGGSGGGGSGGGGSGPA**E**
LPTCSCLDRVIQKDKGPYYTHLGAGPSVAAVREIMENRYGQKGNAIRIEIVVYTGKEGKSSHGCPIAKW
VLRRSSDEEKVLCLVRQRTGHHCPTAVMVVLIMVWDGIPLPMADRLYTELTENLKSYNGHPTDRRCTL
NENRTCTCQGIDPETCGASFSFGCSWSMYFNGCKFGRSPSPRRFRIDPSSPLHEKNLEDNLQSLATRL
APIYKQYAPVAYQNQVEYENVARECRLGSKEGRPFSGVTACLDFCAHPHRDIHNMNNGSTVVCTLTRE
DNRSLGVIPQDEQLHVLPLYKLSDTDEFGSKEGMEAKIKSGAIEVLAPRRKKRTCFTQPVPRSGKKRAA
MMTEVLAHKIRAVEKKPIPRIKRKNNSTTTNNSKPSSLPTLGSNTETVQPEVKSETEPHFILKSSDNTKTY
SLMPSAPHPVKEASPGFSWSPKTASATPAPLKNDATASCGFSERSSTPHCTMPSGRLSGANAAAADG
PGISQLGEVAPLPTLSAPVMEPLINSEPSTGVTEPLTPHQPNHQPSFLTSPQDLASSPMEEDEQHSEAD
EPPSDEPLSDDPLSPAEEKLPHIDEYWSDSEHIFLDANIGGVAIAPAHGSVLIECARRELHATTPVEHPN
RNHPTRLSLVFYQHKNLNKPQHGFELNKIKFEAKEAKNKKMKASEQKDQAANEGPEQSSEVNELNQIP
SHKALTLTHDNVVTVSPYALTHVAGPYNHWVID

Name: **TALE_B-TET1(CD)**

Keys: NLS, TALE-B **TET1(1418-2136),** His tag

MHHHHHHIDGGGGSDPKKKRKVDPKKKRKVDPKKKRKVGSTGSRNDGGGGSGGGGSGGGGSGRAV
DLRTLGYSQQQQEKIKPKVRSTVAQHHEALVGHGFTHAHIVALSQHPAALGTVAVTYQHIITALPEATHED
IVGVGKQWSGARALEALLTDAGELRGPPLQLDTGQLVKIAKRGGVTAMEAVHASRNALTGAPLNLTPDQ
VVAIASNIGGKQALETVQRLLPVLCQDHGLTPDQVVAIASNNGGKQALETVQRLLPVLCQDHGLTPDQVV
AIASHDGGKQALETVQRLLPVLCQDHGLTPDQVVAIASNNGGKQALETVQRLLPVLCQDHGLTPDQVVAI
ASNNGGKQALETVQRLLPVLCQDHGLTPDQVVAIASNNGGKQALETVQRLLPVLCQDHGLTPDQVVAIA
SHDGGKQALETVQRLLPVLCQDHGLTPDQVVAIASNIGGKQALETVQRLLPVLCQDHGLTPDQVVAIASN
NGGKQALETVQRLLPVLCQDHGLTPDQVVAIASNGGGKQALETVQRLLPVLCQDHGLTPDQVVAIASNIG
GKQALETVQRLLPVLCQDHGLTPDQVVAIASNNGGKQALETVQRLLPVLCQDHGLTPDQVVAIASHDGG
KQALETVQRLLPVLCQDHGLTPDQVVAIASHDGGKQALETVQRLLPVLCQDHGLTPDQVVAIASNNGGK
QALETVQRLLPVLCQDHGLTPDQVVAIASHDGGKQALETVQRLLPVLCQDHGLTPDQVVAIASNGGGKQ
ALETVQRLLPVLCQDHGLTPDQVVAIASNGGGKQALESIVAQLSRPDPALAALTNDHLVALACLGGRPAM
DAVKKGLPHAPELIRRVNRRIGERTSHRVARDPKKKRKVDPKKKRKVGGRGGGGSGGGGSGGGGSGP
AELPTCSCLDRVIQKDKGPYYTHLGAGPSVAAVREIMENRYGQKGNAIRIEIVVYTGKEGKSSHGCPIAK
WVLRRSSDEEKVLCLVRQRTGHHCPTAVMVVLIMVWDGIPLPMADRLYTELTENLKSYNGHPTDRRCT
LNENRTCTCQGIDPETCGASFSFGCSWSMYFNGCKFGRSPSPRRFRIDPSSPLHEKNLEDNLQSLATR
LAPIYKQYAPVAYQNQVEYENVARECRLGSKEGRPFSGVTACLDFCAHPHRDIHNMNNGSTVVCTLTR
EDNRSLGVIPQDEQLHVLPLYKLSDTDEFGSKEGMEAKIKSGAIEVLAPRRKKRTCFTQPVPRSGKKRA
AMMTEVLAHKIRAVEKKPIPRIKRKNNSTTTNNSKPSSLPTLGSNTETVQPEVKSETEPHFILKSSDNTK
TYSLMPSAPHPVKEASPGFSWSPKTASATPAPLKNDATASCGFSERSSTPHCTMPSGRLSGANAAAA
DGPGISQLGEVAPLPTLSAPVMEPLINSEPSTGVTEPLTPHQPNHQPSFLTSPQDLASSPMEEDEQHSE
ADEPPSDEPLSDDPLSPAEEKLPHIDEYWSDSEHIFLDANIGGVAIAPAHGSVLIECARRELHATTPVEH
PNRNHPTRLSLVFYQHKNLNKPQHGFELNKIKFEAKEAKNKKMKASEQKDQAANEGPEQSSEVNELN
QIPSHKALTLTHDNVVTVSPYALTHVAGPYNHWVID

Name: **TALE_D-TET1(CD)**

Keys: NLS, TALE_D **TET1(1418-2136),** His tag

MHHHHHHIDGGGGSDPKKKRKVDPKKKRKVDPKKKRKVGSTGSRNDGGGGSGGGGSGGGGSGRAV
DLRTLGYSQQQQEKIKPKVRSTVAQHHEALVGHGFTHAHIVALSQHPAALGTVAVTYQHIITALPEATHED
IVGVGKQWSGARALEALLTDAGELRGPPLQLDTGQLVKIAKRGGVTAMEAVHASRNALTGAPLNLTPDQ
VVAIASNIGGKQALETVQRLLPVLCQDHGLTPDQVVAIASNIGGKQALETVQRLLPVLCQDHGLTPDQVVA
IASNIGGKQALETVQRLLPVLCQDHGLTPDQVVAIASNIGGKQALETVQRLLPVLCQDHGLTPDQVVAIAS
NIGGKQALETVQRLLPVLCQDHGLTPDQVVAIASHDGGKQALETVQRLLPVLCQDHGLTPDQVVAIASNN
GGKQALETVQRLLPVLCQDHGLTPDQVVAIASNIGGKQALETVQRLLPVLCQDHGLTPDQVVAIASNIGG
KQALETVQRLLPVLCQDHGLTPDQVVAIASHDGGKQALETVQRLLPVLCQDHGLTPDQVVAIASHDGGK
QALETVQRLLPVLCQDHGLTPDQVVAIASNIGGKQALETVQRLLPVLCQDHGLTPDQVVAIASNIGGKQAL
ETVQRLLPVLCQDHGLTPDQVVAIASNGGGKQALETVQRLLPVLCQDHGLTPDQVVAIASNIGGKQALET
VQRLLPVLCQDHGLTPDQVVAIASNNGGKQALETVQRLLPVLCQDHGLTPDQVVAIASNNGGKQALETV
QRLLPVLCQDHGLTPDQVVAIASNIGGKQALESIVAQLSRPDPALAALTNDHLVALACLGGRPAMDAVKK
GLPHAPELIRRVNRRIGERTSHRVARDPKKKRKVDPKKKRKVGGRGGGGSGGGGSGGGGSGPAELPT
CSCLDRVIQKDKGPYYTHLGAGPSVAAVREIMENRYGQKGNAIRIEIVVYTGKEGKSSHGCPIAKWVLR
RSSDEEKVLCLVRQRTGHHCPTAVMVVLIMVWDGIPLPMADRLYTELTENLKSYNGHPTDRRCTLNEN
RTCTCQGIDPETCGASFSFGCSWSMYFNGCKFGRSPSPRRFRIDPSSPLHEKNLEDNLQSLATRLAPIY
KQYAPVAYQNQVEYENVARECRLGSKEGRPFSGVTACLDFCAHPHRDIHNMNNGSTVVCTLTREDNR
SLGVIPQDEQLHVLPLYKLSDTDEFGSKEGMEAKIKSGAIEVLAPRRKKRTCFTQPVPRSGKKRAAMM
TEVLAHKIRAVEKKPIPRIKRKNNSTTTNNSKPSSLPTLGSNTETVQPEVKSETEPHFILKSSDNTKTYSL
MPSAPHPVKEASPGFSWSPKTASATPAPLKNDATASCGFSERSSTPHCTMPSGRLSGANAAAADGP
GISQLGEVAPLPTLSAPVMEPLINSEPSTGVTEPLTPHQPNHQPSFLTSPQDLASSPMEEDEQHSEADE
PPSDEPLSDDPLSPAEEKLPHIDEYWSDSEHIFLDANIGGVAIAPAHGSVLIECARRELHATTPVEHPNR
NHPTRLSLVFYQHKNLNKPQHGFELNKIKFEAKEAKNKKMKASEQKDQAANEGPEQSSEVNELNQIPS
HKALTLTHDNVVTVSPYALTHVAGPYNHWVID

Name: **TALE_F-TET1(CD)**

Keys: NLS, TALE_F **TET1(1418-2136)**, His tag

MHHHHHHIDGGGGSDPKKKRKVDPKKKRKVDPKKKRKVGSTGSRNDGGGGSGGGGSGGGGSGRAV
DLRTLGYSQQQQEKIKPKVRSTVAQHHEALVGHGFTHAHIVALSQHPAALGTVAVTYQHIITALPEATHED
IVGVGKQWSGARALEALLTDAGELRGPPLQLDTGQLVKIAKRGGVTAMEAVHASRNALTGAPLNLTPDQ
VVAIASNNGGKQALETVQRLLPVLCQDHGLTPDQVVAIASNIGGKQALETVQRLLPVLCQDHGLTPDQVV
AIASHDGGKQALETVQRLLPVLCQDHGLTPDQVVAIASNNGGKQALETVQRLLPVLCQDHGLTPDQVVAI
ASHDGGKQALETVQRLLPVLCQDHGLTPDQVVAIASNIGGKQALETVQRLLPVLCQDHGLTPDQVVAIAS
NNGGKQALETVQRLLPVLCQDHGLTPDQVVAIASNIGGKQALETVQRLLPVLCQDHGLTPDQVVAIASHD
GGKQALETVQRLLPVLCQDHGLTPDQVVAIASNNGGKQALETVQRLLPVLCQDHGLTPDQVVAIASHDG
GKQALETVQRLLPVLCQDHGLTPDQVVAIASNGGGKQALETVQRLLPVLCQDHGLTPDQVVAIASHDGG
KQALETVQRLLPVLCQDHGLTPDQVVAIASHDGGKQALETVQRLLPVLCQDHGLTPDQVVAIASNIGGKQ
ALETVQRLLPVLCQDHGLTPDQVVAIASHDGGKQALETVQRLLPVLCQDHGLTPDQVVAIASHDGGKQAL
ETVQRLLPVLCQDHGLTPDQVVAIASNIGGKQALESIVAQLSRPDPALAALTNDHLVALACLGGRPAMDA
VKKGLPHAPELIRRVNRRIGERTSHRVARDPKKKRKVDPKKKRKVGGRGGGGSGGGGSGGGGSGPAE
LPTCSCLDRVIQKDKGPYYTHLGAGPSVAAVREIMENRYGQKGNAIRIEIVVYTGKEGKSSHGCPIAKW
VLRRSSDEEKVLCLVRQRTGHHCPTAVMVVLIMVWDGIPLPMADRLYTELTENLKSYNGHPTDRRCTL
NENRTCTCQGIDPETCGASFSFGCSWSMYFNGCKFGRSPSPRRFRIDPSSPLHEKNLEDNLQSLATRL
APIYKQYAPVAYQNQVEYENVARECRLGSKEGRPFSGVTACLDFCAHPHRDIHNMNNGSTVVCTLTRE
DNRSLGVIPQDEQLHVLPLYKLSDTDEFGSKEGMEAKIKSGAIEVLAPRRKKRTCFTQPVPRSGKKRAA
MMTEVLAHKIRAVEKKPIPRIKRKNNSTTTNNSKPSSLPTLGSNTETVQPEVKSETEPHFILKSSDNTKTY
SLMPSAPHPVKEASPGFSWSPKTASATPAPLKNDATASCGFSERSSTPHCTMPSGRLSGANAAAADG
PGISQLGEVAPLPTLSAPVMEPLINSEPSTGVTEPLTPHQPNHQPSFLTSPQDLASSPMEEDEQHSEAD
EPPSDEPLSDDPLSPAEEKLPHIDEYWSDSEHIFLDANIGGVAIAPAHGSVLIECARRELHATTPVEHPN
RNHPTRLSLVFYQHKNLNKPQHGFELNKIKFEAKEAKNKKMKASEQKDQAANEGPEQSSEVNELNQIP
SHKALTLTHDNVVTVSPYALTHVAGPYNHWVID

Name: **PUFa-dTET1(CD) (dead TET1(CD))**

Keys: NLS, PUFa, dTET1(1418-2136 (**H1671Y**, **D1673A**))

MIDGGGGSDPKKKRKVDPKKKRKVDPKKKRKVGSTGSRNDGGGGSGGGGSGGGGSGRAGILPPKKKR
KVSRGRSRLLEDFRNNRYPNLQLREIAGHIMEFSQDQHGSRFIQLKLERATPAERQLVFNEILQAAYQLM
VDVFGNYVIQKFFEFGSLEQKLALAERIRGHVLSLALQMYGSRVIEKALEFIPSDQQNEMVRELDGHVLKC
VKDQNGNHVVQKCIECVQPQSLQFIIDAFKGQVFALSTHPYGCRVIQRILEHCLPDQTLPILEELHQHTEQL
VQDQYGNYVIQHVLEHGRPEDKSKIVAEIRGNVLVLSQHKFASNVVEKCVTHASRTERAVLIDEVCTMND
GPHSALYTMMKDQYANYVVQKMIDVAEPGQRKIVMHKIRPHIATLRKYTYGKHILAKLEKYYMKNGVDLG
DPKKKRKVDPKKKRKVGGRGGGGSGGGGSGGGGSGPAELPTCSCLDRVIQKDKGPYYTHLGAGPSVA
AVREIMENRYGQKGNAIRIEIVVYTGKEGKSSHGCPIAKWVLRRSSDEEKVLCLVRQRTGHHCPTAVMVV
LIMVWDGIPLPMADRLYTELTENLKSYNGHPTDRRCTLNENRTCTCQGIDPETCGASFSFGCSWSMYFN
GCKFGRSPSPRRFRIDPSSPLHEKNLEDNLQSLATRLAPIYKQYAPVAYQNQVEYENVARECRLGSKEG
RPFSGVTACLDFCAHPYRAIHNMNNGSTVVCTLTREDNRSLGVIPQDEQLHVLPLYKLSDTDEFGSKEG
MEAKIKSGAIEVLAPRRKKRTCFTQPVPRSGKKRAAMMTEVLAHKIRAVEKKPIPRIKRKNNSTTTNNSKP
SSLPTLGSNTETVQPEVKSETEPHFILKSSDNTKTYSLMPSAPHPVKEASPGFSWSPKTASATPAPLKND
ATASCGFSERSSTPHCTMPSGRLSGANAAAADGPGISQLGEVAPLPTLSAPVMEPLINSEPSTGVTEPLT
PHQPNHQPSFLTSPQDLASSPMEEDEQHSEADEPPSDEPLSDDPLSPAEEKLPHIDEYWSDSEHIFLDA
NIGGVAIAPAHGSVLIECARRELHATTPVEHPNRNHPTRLSLVFYQHKNLNKPQHGFELNKIKFEAKEAKN
KKMKASEQKDQAANEGPEQSSEVNELNQIPSHKALTLTHDNVVTVSPYALTHVAGPYNHWVID

Name: **PUFa-hGADD45A-TET1(CD)**

Keys: NLS, PUFa, GADD45A, **TET1(1418-2136)**

MIDGGGGSDPKKKRKVDPKKKRKVDPKKKRKVGSTGSRNDGGGGSGGGGSGGGGSGRAGILPPKKKR
KVSRGRSRLLEDFRNNRYPNLQLREIAGHIMEFSQDQHGSRFIQLKLERATPAERQLVFNEILQAAYQLM
VDVFGNYVIQKFFEFGSLEQKLALAERIRGHVLSLALQMYGSRVIEKALEFIPSDQQNEMVRELDGHVLKC

VKDQNGNHVVQKCIECVQPQSLQFIIDAFKGQVFALSTHPYGCRVIQRILEHCLPDQTLPILEELHQHTEQL
VQDQYGNYVIQHVLEHGRPEDKSKIVAEIRGNVLVLSQHKFASNVVEKCVTHASRTERAVLIDEVCTMND
GPHSALYTMMKDQYANYVVQKMIDVAEPGQRKIVMHKIRPHIATLRKYTYGKHILAKLEKYYMKNGVDLG
DPKKKRKVDPKKKRKVGGRGGGSGGGSGGGSGGGSGGGSGGGSLTLEEFSAGEQKTERMDKVGDAL
EEVLSKALSQRTITVGVYEAAKLLNVDPDNVVLCLLAADEDDDRDVALQIHFTLIQAFCCENDINILRVSNP
GRLAELLLLETDAGPAASEGAEQPPDLHCVLVTNPHSSQWKDPALSQLICFCRESRYMDQWVPVINLPE
RSRGRGGGGSGGGGSGGGGSGPA**ELPTCSCLDRVIQKDKGPYYTHLGAGPSVAAVREIMENRYGQK**
GNAIRIEIVVYTGKEGKSSHGCPIAKWVLRRSSDEEKVLCLVRQRTGHHCPTAVMVVLIMVWDGIPLPM
ADRLYTELTENLKSYNGHPTDRRCTLNENRTCTCQGIDPETCGASFSFGCSWSMYFNGCKFGRSPSPR
RFRIDPSSPLHEKNLEDNLQSLATRLAPIYKQYAPVAYQNQVEYENVARECRLGSKEGRPFSGVTACLD
FCAHPHRDIHNMNNGSTVVCTLTREDNRSLGVIPQDEQLHVLPLYKLSDTDEFGSKEGMEAKIKSGAIE
VLAPRRKKRTCFTQPVPRSGKKRAAMMTEVLAHKIRAVEKKPIPRIKRKNNSTTTNNSKPSSLPTLGSN
TETVQPEVKSETEPHFILKSSDNTKTYSLMPSAPHPVKEASPGFSWSPKTASATPAPLKNDATASCGFS
ERSSTPHCTMPSGRLSGANAAAADGPGISQLGEVAPLPTLSAPVMEPLINSEPSTGVTEPLTPHQPNH
QPSFLTSPQDLASSPMEEDEQHSEADEPPSDEPLSDDPLSPAEEKLPHIDEYWSDSEHIFLDANIGGVAI
APAHGSVLIECARRELHATTPVEHPNRNHPTRLSLVFYQHKNLNKPQHGFELNKIKFEAKEAKNKKMK
ASEQKDQAANEGPEQSSEVNELNQIPSHKALTLTHDNVVTVSPYALTHVAGPYNHWVID

Name: **hGADD45A-PUFa-TET1(CD)**

Keys: NLS, PUFa, GADD45A, **TET1(1418-2136)**

MTLEEFSAGEQKTERMDKVGDALEEVLSKALSQRTITVGVYEAAKLLNVDPDNVVLCLLAADEDDDRDVA
LQIHFTLIQAFCCENDINILRVSNPGRLAELLLLETDAGPAASEGAEQPPDLHCVLVTNPHSSQWKDPALS
QLICFCRESRYMDQWVPVINLPERSRTGAATMIDGGGGSDPKKKRKVDPKKKRKVDPKKKRKVGSTGS
RNDGGGGSGGGGSGGGGSGRAGILPPKKKRKVSRGRSRLLEDFRNNRYPNLQLREIAGHIMEFSQDQH
GSRFIQLKLERATPAERQLVFNEILQAAYQLMVDVFGNYVIQKFFEFGSLEQKLALAERIRGHVLSLALQM
YGSRVIEKALEFIPSDQQNEMVRELDGHVLKCVKDQNGNHVVQKCIECVQPQSLQFIIDAFKGQVFALST
HPYGCRVIQRILEHCLPDQTLPILEELHQHTEQLVQDQYGNYVIQHVLEHGRPEDKSKIVAEIRGNVLVLS
QHKFASNVVEKCVTHASRTERAVLIDEVCTMNDGPHSALYTMMKDQYANYVVQKMIDVAEPGQRKIVMH
KIRPHIATLRKYTYGKHILAKLEKYYMKNGVDLGDPKKKRKVDPKKKRKVGGRGGGGSGGGGSGGGGS
GPA**ELPTCSCLDRVIQKDKGPYYTHLGAGPSVAAVREIMENRYGQKGNAIRIEIVVYTGKEGKSSHGCPI**
AKWVLRRSSDEEKVLCLVRQRTGHHCPTAVMVVLIMVWDGIPLPMADRLYTELTENLKSYNGHPTDRR
CTLNENRTCTCQGIDPETCGASFSFGCSWSMYFNGCKFGRSPSPRRFRIDPSSPLHEKNLEDNLQSLA
TRLAPIYKQYAPVAYQNQVEYENVARECRLGSKEGRPFSGVTACLDFCAHPHRDIHNMNNGSTVVCTL
TREDNRSLGVIPQDEQLHVLPLYKLSDTDEFGSKEGMEAKIKSGAIEVLAPRRKKRTCFTQPVPRSGKK
RAAMMTEVLAHKIRAVEKKPIPRIKRKNNSTTTNNSKPSSLPTLGSNTETVQPEVKSETEPHFILKSSDN
TKTYSLMPSAPHPVKEASPGFSWSPKTASATPAPLKNDATASCGFSERSSTPHCTMPSGRLSGANAA
AADGPGISQLGEVAPLPTLSAPVMEPLINSEPSTGVTEPLTPHQPNHQPSFLTSPQDLASSPMEEDEQH
SEADEPPSDEPLSDDPLSPAEEKLPHIDEYWSDSEHIFLDANIGGVAIAPAHGSVLIECARRELHATTPV
EHPNRNHPTRLSLVFYQHKNLNKPQHGFELNKIKFEAKEAKNKKMKASEQKDQAANEGPEQSSEVNE
LNQIPSHKALTLTHDNVVTVSPYALTHVAGPYNHWVID

Name: **PUFc-hGADD45A**

Keys: NLS, PUFc, GADD45A, HA tag

MIDGGGGSDPKKKRKVDPKKKRKVDPKKKRKVGSTGSRNDGGGGSGGGGSGGGGSGRAGILPPKKKR
KVSRGRSRLLEDFRNNRYPNLQLREIAGHIMEFSQDQHGSRFIQLKLERATPAERQLVFNEILQAAYQLM
VDVFGNYVIQKFFEFGSLEQKLALAERIRGHVLSLALQMYGSRVIEKALEFIPSDQQNEMVRELDGHVLKC
VKDQNGNHVVQKCIECVQPQSLQFIIDAFKGQVFALSTHPYGCRVIQRILEHCLPDQTLPILEELHQHTEQL
VQDQYGSYVIEHVLEHGRPEDKSKIVAEIRGNVLVLSQHKFANNVVQKCVTHASRTERAVLIDEVCTMND
GPHSALYTMMKDQYANYVVQKMIDVAEPGQRKIVMHKIRPHIATLRKYTYGKHILAKLEKYYMKNGVDLG
DPKKKRKVDPKKKRKVGGRGGGGSGGGGSGGGGSGPALTLEEFSAGEQKTERMDKVGDALEEVLSKA
LSQRTITVGVYEAAKLLNVDPDNVVLCLLAADEDDDRDVALQIHFTLIQAFCCENDINILRVSNPGRLAELLL
LETDAGPAASEGAEQPPDLHCVLVTNPHSSQWKDPALSQLICFCRESRYMDQWVPVINLPERSRYPYDV
PDYA

Name: **hGADD45A-PUFc**

Keys: NLS, PUFc, GADD45A, HA tag

MTLEEFSAGEQKTERMDKVGDALEEVLSKALSQRTITVGVYEAAKLLNVDPDNVVLCLLAADEDDDRDVA
LQIHFTLIQAFCCENDINILRVSNPGRLAELLLLETDAGPAASEGAEQPPDLHCVLVTNPHSSQWKDPALS
QLICFCRESRYMDQWVPVINLPERSRYPYDVPDYAIDGGGGSDPKKKRKVDPKKKRKVDPKKKRKVGST
GSRNDGGGGSGGGGSGGGGSGRAGILPPKKKRKVSRGRSRLLEDFRNNRYPNLQLREIAGHIMEFSQD
QHGSRFIQLKLERATPAERQLVFNEILQAAYQLMVDVFGNYVIQKFFEFGSLEQKLALAERIRGHVLSLAL
QMYGSRVIEKALEFIPSDQQNEMVRELDGHVLKCVKDQNGNHVVQKCIECVQPQSLQFIIDAFKGQVFAL
STHPYGCRVIQRILEHCLPDQTLPILEELHQHTEQLVQDQYGSYVIEHVLEHGRPEDKSKIVAEIRGNVLVL
SQHKFANNVVQKCVTHASRTERAVLIDEVCTMNDGPHSALYTMMKDQYANYVVQKMIDVAEPGQRKIV
MHKIRPHIATLRKYTYGKHILAKLEKYYMKNGVDLGDPKKKRKVDPKKKRKVGGRGGGGSGGGGSGGG
GSGPA

Name: **hNEIL2-PUFa-TET1(CD)**

Keys: NLS, PUFa, NEIL2, **TET1(1418-2136), Flag tag**

M**DYKDDDDK**PKKKRKLPEGPLVRKFHHLVSPFVGQQVVKTGGSSKKLQPASLQSLWLQDTQVHGKKLF
LRFDLDEEMGPPGSSPTPEPPQKEVQKEGAADPKQVGEPSGQKTLDGSSRSAELVPQGEDDSEYLERD
APAGDAGRWLRVSFGLFGSVWVNDFSRAKKANKRGDWRDPSPRLVLHFGGGGFLAFYNCQLSWSSSP
VVTPTCDILSEKFHRGQALEALGQAQPVCYTLLDQRYFSGLGNIIKNEALYRAGIHPLSLGSVLSASRREVL
VDHVVEFSTAWLQGKFQGRPQHTQVYQKEQCPAGHQVMKEAFGPEDGLQRLTWWCPQCQPQLSEEP
EQCQFSGAATMIDGGGGSDPKKKRKVDPKKKRKVDPKKKRKVGSTGSRNDGGGGSGGGGSGGGGSG
RAGILPPKKKRKVSRGRSRLLEDFRNNRYPNLQLREIAGHIMEFSQDQHGSRFIQLKLERATPAERQLVF
NEILQAAYQLMVDVFGNYVIQKFFEFGSLEQKLALAERIRGHVLSLALQMYGSRVIEKALEFIPSDQQNEM
VRELDGHVLKCVKDQNGNHVVQKCIECVQPQSLQFIIDAFKGQVFALSTHPYGCRVIQRILEHCLPDQTLP
ILEELHQHTEQLVQDQYGNYVIQHVLEHGRPEDKSKIVAEIRGNVLVLSQHKFASNVVEKCVTHASRTERA
VLIDEVCTMNDGPHSALYTMMKDQYANYVVQKMIDVAEPGQRKIVMHKIRPHIATLRKYTYGKHILAKLEK
YYMKNGVDLGDPKKKRKVDPKKKRKVGGRGGGGSGGGGSGGGGSGPA**ELPTCSCLDRVIQKDKGPY
YTHLGAGPSVAAVREIMENRYGQKGNAIRIEIVVYTGKEGKSSHGCPIAKWVLRRSSDEEKVLCLVRQR
TGHHCPTAVMVVLIMVWDGIPLPMADRLYTELTENLKSYNGHPTDRRCTLNENRTCTCQGIDPETCGAS
FSFGCSWSMYFNGCKFGRSPSPRRFRIDPSSPLHEKNLEDNLQSLATRLAPIYKQYAPVAYQNQVEYE
NVARECRLGSKEGRPFSGVTACLDFCAHPHRDIHNMNNGSTVVCTLTREDNRSLGVIPQDEQLHVLPL
YKLSDTDEFGSKEGMEAKIKSGAIEVLAPRRKKRTCFTQPVPRSGKKRAAMMTEVLAHKIRAVEKKPIP
RIKRKNNSTTTNNSKPSSLPTLGSNTETVQPEVKSETEPHFILKSSDNTKTYSLMPSAPHPVKEASPGFS
WSPKTASATPAPLKNDATASCGFSERSSTPHCTMPSGRLSGANAAAADGPGISQLGEVAPLPTLSAPV
MEPLINSEPSTGVTEPLTPHQPNHQPSFLTSPQDLASSPMEEDEQHSEADEPPSDEPLSDDPLSPAEEK
LPHIDEYWSDSEHIFLDANIGGVAIAPAHGSVLIECARRELHATTPVEHPNRNHPTRLSLVFYQHKNLNK
PQHGFELNKIKFEAKEAKNKKMKASEQKDQAANEGPEQSSEVNELNQIPSHKALTLTHDNVVTVSPYA
LTHVAGPYNHWV**ID

Name: **PUFa-hNEIL2-TET1(CD)**

Keys: NLS, PUFa, NEIL2, **TET1(1418-2136)**

MIDGGGGSDPKKKRKVDPKKKRKVDPKKKRKVGSTGSRNDGGGGSGGGGSGGGGSGRAGILPPKKKR
KVSRGRSRLLEDFRNNRYPNLQLREIAGHIMEFSQDQHGSRFIQLKLERATPAERQLVFNEILQAAYQLM
VDVFGNYVIQKFFEFGSLEQKLALAERIRGHVLSLALQMYGSRVIEKALEFIPSDQQNEMVRELDGHVLKC
VKDQNGNHVVQKCIECVQPQSLQFIIDAFKGQVFALSTHPYGCRVIQRILEHCLPDQTLPILEELHQHTEQL
VQDQYGNYVIQHVLEHGRPEDKSKIVAEIRGNVLVLSQHKFASNVVEKCVTHASRTERAVLIDEVCTMND
GPHSALYTMMKDQYANYVVQKMIDVAEPGQRKIVMHKIRPHIATLRKYTYGKHILAKLEKYYMKNGVDLG
DPKKKRKVDPKKKRKVGGRGGGSGGGSGGGSGGGSGGGSGGGSLPEGPLVRKFHHLVSPFVGQQVV
KTGGSSKKLQPASLQSLWLQDTQVHGKKLFLRFDLDEEMGPPGSSPTPEPPQKEVQKEGAADPKQVGE
PSGQKTLDGSSRSAELVPQGEDDSEYLERDAPAGDAGRWLRVSFGLFGSVWVNDFSRAKKANKRGDW
RDPSPRLVLHFGGGGFLAFYNCQLSWSSSPVVTPTCDILSEKFHRGQALEALGQAQPVCYTLLDQRYFS
GLGNIIKNEALYRAGIHPLSLGSVLSASRREVLVDHVVEFSTAWLQGKFQGRPQHTQVYQKEQCPAGHQ

VMKEAFGPEDGLQRLTWWCPQCQPQLSEEPEQCQFSRGGGGSGGGGSGGGGSGPA**ELPTCSCLDR<br>VIQKDKGPYYTHLGAGPSVAAVREIMENRYGQKGNAIRIEIVVYTGKEGKSSHGCPIAKWVLRRSSDEE<br>KVLCLVRQRTGHHCPTAVMVVLIMVWDGIPLPMADRLYTELTENLKSYNGHPTDRRCTLNENRTCTCQ<br>GIDPETCGASFSFGCSWSMYFNGCKFGRSPSPRRFRIDPSSPLHEKNLEDNLQSLATRLAPIYKQYAPV<br>AYQNQVEYENVARECRLGSKEGRPFSGVTACLDFCAHPHRDIHNMNNGSTVVCTLTREDNRSLGVIPQ<br>DEQLHVLPLYKLSDTDEFGSKEGMEAKIKSGAIEVLAPRRKKRTCFTQPVPRSGKKRAAMMTEVLAHKI<br>RAVEKKPIPRIKRKNNSTTTNNSKPSSLPTLGSNTETVQPEVKSETEPHFILKSSDNTKTYSLMPSAPHP<br>VKEASPGFSWSPKTASATPAPLKNDATASCGFSERSSTPHCTMPSGRLSGANAAAADGPGISQLGEV<br>APLPTLSAPVMEPLINSEPSTGVTEPLTPHQPNHQPSFLTSPQDLASSPMEEDEQHSEADEPPSDEPLS<br>DDPLSPAEEKLPHIDEYWSDSEHIFLDANIGGVAIAPAHGSVLIECARRELHATTPVEHPNRNHPTRLSL<br>VFYQHKNLNKPQHGFELNKIKFEAKEAKNKKMKASEQKDQAANEGPEQSSEVNELNQIPSHKALTLTH<br>DNVVTVSPYALTHVAGPYNHWV**ID
Name: **PUFc-hNEIL2**
**Keys:** NLS, PUFc, NEIL2
MIDGGGGSDPKKKRKVDPKKKRKVDPKKKRKVGSTGSRNDGGGGSGGGGSGGGGSGRAGILPPKKKR<br>KVSRGRSRLLEDFRNNRYPNLQLREIAGHIMEFSQDQHGSRFIQLKLERATPAERQLVFNEILQAAYQLM<br>VDVFGNYVIQKFFEFGSLEQKLALAERIRGHVLSLALQMYGSRVIEKALEFIPSDQQNEMVRELDGHVLKC<br>VKDQNGNHVVQKCIECVQPQSLQFIIDAFKGQVFALSTHPYGCRVIQRILEHCLPDQTLPILEELHQHTEQL<br>VQDQYGSYVIEHVLEHGRPEDKSKIVAEIRGNVLVLSQHKFANNVVQKCVTHASRTERAVLIDEVCTMND<br>GPHSALYTMMKDQYANYVVQKMIDVAEPGQRKIVMHKIRPHIATLRKYTYGKHILAKLEKYYMKNGVDLG<br>DPKKKRKVDPKKKRKVGGRGGGGSGGGGSGGGGSGPALPEGPLVRKFHHLVSPFVGQQVVKTGGSS<br>KKLQPASLQSLWLQDTQVHGKKLFLRFDLDEEMGPPGSSPTPEPPQKEVQKEGAADPKQVGEPSGQKT<br>LDGSSRSAELVPQGEDDSEYLERDAPAGDAGRWLRVSFGLFGSVWVNDFSRAKKANKRGDWRDPSPR<br>LVLHFGGGGFLAFYNCQLSWSSSPVVTPTCDILSEKFHRGQALEALGQAQPVCYTLLDQRYFSGLGNIIK<br>NEALYRAGIHPLSLGSVLSASRREVLVDHVVEFSTAWLQGKFQGRPQHTQVYQKEQCPAGHQVMKEAF<br>GPEDGLQRLTWWCPQCQPQLSEEPEQCQFS
Name: **hNEIL2- PUFc**
**Keys:** NLS, PUFc, NEIL2
MPEGPLVRKFHHLVSPFVGQQVVKTGGSSKKLQPASLQSLWLQDTQVHGKKLFLRFDLDEEMGPPGSS<br>PTPEPPQKEVQKEGAADPKQVGEPSGQKTLDGSSRSAELVPQGEDDSEYLERDAPAGDAGRWLRVSF<br>GLFGSVWVNDFSRAKKANKRGDWRDPSPRLVLHFGGGGFLAFYNCQLSWSSSPVVTPTCDILSEKFHR<br>GQALEALGQAQPVCYTLLDQRYFSGLGNIIKNEALYRAGIHPLSLGSVLSASRREVLVDHVVEFSTAWLQ<br>GKFQGRPQHTQVYQKEQCPAGHQVMKEAFGPEDGLQRLTWWCPQCQPQLSEEPEQCQFSIDGGGGS<br>DPKKKRKVDPKKKRKVDPKKKRKVGSTGSRNDGGGGSGGGGSGGGGSGRAGILPPKKKRKVSRGRSR<br>LLEDFRNNRYPNLQLREIAGHIMEFSQDQHGSRFIQLKLERATPAERQLVFNEILQAAYQLMVDVFGNYVI<br>QKFFEFGSLEQKLALAERIRGHVLSLALQMYGSRVIEKALEFIPSDQQNEMVRELDGHVLKCVKDQNGNH<br>VVQKCIECVQPQSLQFIIDAFKGQVFALSTHPYGCRVIQRILEHCLPDQTLPILEELHQHTEQLVQDQYGSY<br>VIEHVLEHGRPEDKSKIVAEIRGNVLVLSQHKFANNVVQKCVTHASRTERAVLIDEVCTMNDGPHSALYT<br>MMKDQYANYVVQKMIDVAEPGQRKIVMHKIRPHIATLRKYTYGKHILAKLEKYYMKNGVDLGDPKKKRKV<br>DPKKKRKVGGRGGGGSGGGGSGGGGSGPA
Name: **hNEIL1-PUFa-TET1(CD)**
**Keys:** NLS, PUFa, NEIL1, **TET1(1418-2136)**, **Flag tag**
M**DYKDDDDK**PKKKRKLPEGPELHLASQFVNEACRALVFGGCVEKSSVSRNPEVPFESSAYRISASARGK<br>ELRLILSPLPGAQPQQEPLALVFRFGMSGSFQLVPREELPRHAHLRFYTAPPGPRLALCFVDIRRFGRWD<br>LGGKWQPGRGPCVLQEYQQFRENVLRNLADKAFDRPICEALLDQRFFNGIGNYLRAEILYRLKIPPFEKA<br>RSVLEALQQHRPSPELTLSQKIRTKLQNPDLLELCHSVPKEVVQLGGRGYGSESGEEDFAAFRAWLRCY<br>GMPGMSSLQDRHGRTIWFQGDPGPLAPKGRKSRKKKSATQLSPEDRVEDALPPSKAPSRTRRAKRDL<br>PKRTATQRPEGTSLQQDPEAPTVPKKGRRKGRQAASGHCRPRKVKADIPSLEPEGTSASGAATMIDGG<br>GGSDPKKKRKVDPKKKRKVDPKKKRKVGSTGSRNDGGGGSGGGGSGGGGSGRAGILPPKKKRKVSR<br>GRSRLLEDFRNNRYPNLQLREIAGHIMEFSQDQHGSRFIQLKLERATPAERQLVFNEILQAAYQLMVDVF

GNYVIQKFFEFGSLEQKLALAERIRGHVLSLALQMYGSRVIEKALEFIPSDQQNEMVRELDGHVLKCVKDQ
NGNHVVQKCIECVQPQSLQFIIDAFKGQVFALSTHPYGCRVIQRILEHCLPDQTLPILEELHQHTEQLVQD
QYGNYVIQHVLEHGRPEDKSKIVAEIRGNVLVLSQHKFASNVVEKCVTHASRTERAVLIDEVCTMNDGPH
SALYTMMKDQYANYVVQKMIDVAEPGQRKIVMHKIRPHIATLRKYTYGKHILAKLEKYYMKNGVDLGDPK
KKRKVDPKKKRKVGGRGGGGSGGGGSGGGGSGPA**ELPTCSCLDRVIQKDKGPYYTHLGAGPSVAAV
REIMENRYGQKGNAIRIEIVVYTGKEGKSSHGCPIAKWVLRRSSDEEKVLCLVRQRTGHHCPTAVMVVLI
MVWDGIPLPMADRLYTELTENLKSYNGHPTDRRCTLNENRTCTCQGIDPETCGASFSFGCSWSMYFNG
CKFGRSPSPRRFRIDPSSPLHEKNLEDNLQSLATRLAPIYKQYAPVAYQNQVEYENVARECRLGSKEG
RPFSGVTACLDFCAHPHRDIHNMNNGSTVVCTLTREDNRSLGVIPQDEQLHVLPLYKLSDTDEFGSKEG
MEAKIKSGAIEVLAPRRKKRTCFTQPVPRSGKKRAAMMTEVLAHKIRAVEKKPIPRIKRKNNSTTTNNS
KPSSLPTLGSNTETVQPEVKSETEPHFILKSSDNTKTYSLMPSAPHPVKEASPGFSWSPKTASATPAPL
KNDATASCGFSERSSTPHCTMPSGRLSGANAAAADGPGISQLGEVAPLPTLSAPVMEPLINSEPSTGV
TEPLTPHQPNHQPSFLTSPQDLASSPMEEDEQHSEADEPPSDEPLSDDPLSPAEEKLPHIDEYWSDSE
HIFLDANIGGVAIAPAHGSVLIECARRELHATTPVEHPNRNHPTRLSLVFYQHKNLNKPQHGFELNKIKF
EAKEAKNKKMKASEQKDQAANEGPEQSSEVNELNQIPSHKALTLTHDNVVTVSPYALTHVAGPYNHW
V**ID

Name: **PUFa-hNEIL1-TET1(CD)**

Keys: NLS, PUFa, NEIL1, **TET1(1418-2136), Flag tag**

MIDGGGGSDPKKKRKVDPKKKRKVDPKKKRKVGSTGSRNDGGGGSGGGGSGGGGSGRAGILPPKKKR
KVSRGRSRLLEDFRNNRYPNLQLREIAGHIMEFSQDQHGSRFIQLKLERATPAERQLVFNEILQAAYQLM
VDVFGNYVIQKFFEFGSLEQKLALAERIRGHVLSLALQMYGSRVIEKALEFIPSDQQNEMVRELDGHVLKC
VKDQNGNHVVQKCIECVQPQSLQFIIDAFKGQVFALSTHPYGCRVIQRILEHCLPDQTLPILEELHQHTEQL
VQDQYGNYVIQHVLEHGRPEDKSKIVAEIRGNVLVLSQHKFASNVVEKCVTHASRTERAVLIDEVCTMND
GPHSALYTMMKDQYANYVVQKMIDVAEPGQRKIVMHKIRPHIATLRKYTYGKHILAKLEKYYMKNGVDLG
DPKKKRKVDPKKKRKVGGRGGGSGGGSGGGSGGGSGGGSGGGSLPEGPELHLASQFVNEACRALVF
GGCVEKSSVSRNPEVPFESSAYRISASARGKELRLILSPLPGAQPQQEPLALVFRFGMSGSFQLVPREEL
PRHAHLRFYTAPPGPRLALCFVDIRRFGRWDLGGKWQPGRGPCVLQEYQQFRENVLRNLADKAFDRPI
CEALLDQRFFNGIGNYLRAEILYRLKIPPFEKARSVLEALQQHRPSPELTLSQKIRTKLQNPDLLELCHSVP
KEVVQLGGRGYGSESGEEDFAAFRAWLRCYGMPGMSSLQDRHGRTIWFQGDPGPLAPKGRKSRKKS
KATQLSPEDRVEDALPPSKAPSRTRRAKRDLPKRTATQRPEGTSLQQDPEAPTVPKKGRRKGRQAASG
HCRPRKVKADIPSLEPEGTSASRGGGGSGGGGSGGGGSGPA**ELPTCSCLDRVIQKDKGPYYTHLGAG
PSVAAVREIMENRYGQKGNAIRIEIVVYTGKEGKSSHGCPIAKWVLRRSSDEEKVLCLVRQRTGHHCPT
AVMVVLIMVWDGIPLPMADRLYTELTENLKSYNGHPTDRRCTLNENRTCTCQGIDPETCGASFSFGCS
WSMYFNGCKFGRSPSPRRFRIDPSSPLHEKNLEDNLQSLATRLAPIYKQYAPVAYQNQVEYENVAREC
RLGSKEGRPFSGVTACLDFCAHPHRDIHNMNNGSTVVCTLTREDNRSLGVIPQDEQLHVLPLYKLSDTD
EFGSKEGMEAKIKSGAIEVLAPRRKKRTCFTQPVPRSGKKRAAMMTEVLAHKIRAVEKKPIPRIKRKNN
STTTNNSKPSSLPTLGSNTETVQPEVKSETEPHFILKSSDNTKTYSLMPSAPHPVKEASPGFSWSPKTA
SATPAPLKNDATASCGFSERSSTPHCTMPSGRLSGANAAAADGPGISQLGEVAPLPTLSAPVMEPLIN
SEPSTGVTEPLTPHQPNHQPSFLTSPQDLASSPMEEDEQHSEADEPPSDEPLSDDPLSPAEEKLPHIDE
YWSDSEHIFLDANIGGVAIAPAHGSVLIECARRELHATTPVEHPNRNHPTRLSLVFYQHKNLNKPQHGF
ELNKIKFEAKEAKNKKMKASEQKDQAANEGPEQSSEVNELNQIPSHKALTLTHDNVVTVSPYALTHVA
GPYNHWV**ID

Name: **hNEIL3-PUFa-TET1(CD)**

Keys: NLS, PUFa, NEIL3, **TET1(1418-2136), Flag tag**

M**DYKDDDDK**PKKKRKLVEGPGCTLNGEKIRARVLPGQAVTGVRGSALRSLQGRALRLAASTVVVSPQA
AALNNDSSQNVLSLFNGYVYSGVETLGKELFMYFGPKALRIHFGMKGFIMINPLEYKYKNGASPVLEVQLT
KDLICFFDSSVELRNSMESQQRIRMMKELDVCSPEFSFLRAESEVKKQKGRMLGDVLMDQNVLPGVGNII
KNEALFDSGLHPAVKVCQLTDEQIHHLMKMIRDFSILFYRCRKAGLALSKHYKVYKRPNCGQCHCRITVC
RFGDNNRMTYFCPHCQKENPQHVDICKLPTRNTIISWTSSRVDHVMDSVARKSEEHWTCVVCTLINKPS
SKACDACLTSRPIDSVLKSEENSTVFSHLMKYPCNTFGKPHTEVKINRKTAFGTTTLVLTDFSNKSSTLER
KTKQNQILDEEFQNSPPASVCLNDIQHPSKKTTNDITQLSSKVNISPTISSESKLFSPAHKKPKTAHYSSPE
LKSCNPGYSNSELQINMTDGPRTLNPDSPRCSKHNRLCILRVVRKDGENKGRQFYACPLPREAQCGFFE

WADLSFPFCNHGKRSTMKTVLKIGPNNGKNFFVCPLGKEKQCNFFQWAENGPGIKIIPGCGAATMIDGG
GGSDPKKKRKVDPKKKRKVDPKKKRKVGSTGSRNDGGGGSGGGGSGGGGSGRAGILPPKKKRKVSR
GRSRLLEDFRNNRYPNLQLREIAGHIMEFSQDQHGSRFIQLKLERATPAERQLVFNEILQAAYQLMVDVF
GNYVIQKFFEFGSLEQKLALAERIRGHVLSLALQMYGSRVIEKALEFIPSDQQNEMVRELDGHVLKCVKDQ
NGNHVVQKCIECVQPQSLQFIIDAFKGQVFALSTHPYGCRVIQRILEHCLPDQTLPILEELHQHTEQLVQD
QYGNYVIQHVLEHGRPEDKSKIVAEIRGNVLVLSQHKFASNVVEKCVTHASRTERAVLIDEVCTMNDGPH
SALYTMMKDQYANYVVQKMIDVAEPGQRKIVMHKIRPHIATLRKYTYGKHILAKLEKYYMKNGVDLGDPK
KKRKVDPKKKRKVGGRGGGGSGGGGSGGGGSGPA**ELPTCSCLDRVIQKDKGPYYTHLGAGPSVAAV**
REIMENRYGQKGNAIRIEIVVYTGKEGKSSHGCPIAKWVLRRSSDEEKVLCLVRQRTGHHCPTAVMVVLI
MVWDGIPLPMADRLYTELTENLKSYNGHPTDRRCTLNENRTCTCQGIDPETCGASFSFGCSWSMYFNG
CKFGRSPSPRRFRIDPSSPLHEKNLEDNLQSLATRLAPIYKQYAPVAYQNQVEYENVARECRLGSKEG
RPFSGVTACLDFCAHPHRDIHNMNNGSTVVCTLTREDNRSLGVIPQDEQLHVLPLYKLSDTDEFGSKEG
MEAKIKSGAIEVLAPRRKKRTCFTQPVPRSGKKRAAMMTEVLAHKIRAVEKKPIPRIKRKNNSTTTNNS
KPSSLPTLGSNTETVQPEVKSETEPHFILKSSDNTKTYSLMPSAPHPVKEASPGFSWSPKTASATPAPL
KNDATASCGFSERSSTPHCTMPSGRLSGANAAAADGPGISQLGEVAPLPTLSAPVMEPLINSEPSTGV
TEPLTPHQPNHQPSFLTSPQDLASSPMEEDEQHSEADEPPSDEPLSDDPLSPAEEKLPHIDEYWSDSE
HIFLDANIGGVAIAPAHGSVLIECARRELHATTPVEHPNRNHPTRLSLVFYQHKNLNKPQHGFELNKIKF
EAKEAKNKKMKASEQKDQAANEGPEQSSEVNELNQIPSHKALTLTHDNVVTVSPYALTHVAGPYNHW
VID

Name: **hTDG-PUFa-TET1(CD)**

Keys: NLS, PUFa, TDG, **TET1(1418-2136), Flag tag**

M**DYKDDDDK**PKKKRKLEAENAGSYSLQQAQAFYTFPFQQLMAEAPNMAVVNEQQMPEEVPAPAPAQE
PVQEAPKGRKRKPRTTEPKQPVEPKKPVESKKSGKSAKSKEKQEKITDTFKVKRKVDRFNGVSEAELLTK
TLPDILTFNLDIVIIGINPGLMAAYKGHHYPGPGNHFWKCLFMSGLSEVQLNHMDDHTLPGKYGIGFTNMV
ERTTPGSKDLSSKEFREGGRILVQKLQKYQPRIAVFNGKCIYEIFSKEVFGVKVKNLEFGLQPHKIPDTETL
CYVMPSSSARCAQFPRAQDKVHYYIKLKDLRDQLKGIERNMDVQEVQYTFDLQLAQEDAKKMAVKEEKY
DPGYEAAYGGAYGENPCSSEPCGFSSNGLIESVELRGESAFSGIPNGQWMTQSFTDQIPSFSNHCGTQ
EQEEESHATGAATMIDGGGGSDPKKKRKVDPKKKRKVDPKKKRKVGSTGSRNDGGGGSGGGGSGGG
GSGRAGILPPKKKRKVSRGRSRLLEDFRNNRYPNLQLREIAGHIMEFSQDQHGSRFIQLKLERATPAERQ
LVFNEILQAAYQLMVDVFGNYVIQKFFEFGSLEQKLALAERIRGHVLSLALQMYGSRVIEKALEFIPSDQQN
EMVRELDGHVLKCVKDQNGNHVVQKCIECVQPQSLQFIIDAFKGQVFALSTHPYGCRVIQRILEHCLPDQ
TLPILEELHQHTEQLVQDQYGNYVIQHVLEHGRPEDKSKIVAEIRGNVLVLSQHKFASNVVEKCVTHASRT
ERAVLIDEVCTMNDGPHSALYTMMKDQYANYVVQKMIDVAEPGQRKIVMHKIRPHIATLRKYTYGKHILAK
LEKYYMKNGVDLGDPKKKRKVDPKKKRKVGGRGGGGSGGGGSGGGGSGPA**ELPTCSCLDRVIQKDK**
GPYYTHLGAGPSVAAVREIMENRYGQKGNAIRIEIVVYTGKEGKSSHGCPIAKWVLRRSSDEEKVLCLV
RQRTGHHCPTAVMVVLIMVWDGIPLPMADRLYTELTENLKSYNGHPTDRRCTLNENRTCTCQGIDPET
CGASFSFGCSWSMYFNGCKFGRSPSPRRFRIDPSSPLHEKNLEDNLQSLATRLAPIYKQYAPVAYQNQ
VEYENVARECRLGSKEGRPFSGVTACLDFCAHPHRDIHNMNNGSTVVCTLTREDNRSLGVIPQDEQLH
VLPLYKLSDTDEFGSKEGMEAKIKSGAIEVLAPRRKKRTCFTQPVPRSGKKRAAMMTEVLAHKIRAVEK
KPIPRIKRKNNSTTTNNSKPSSLPTLGSNTETVQPEVKSETEPHFILKSSDNTKTYSLMPSAPHPVKEAS
PGFSWSPKTASATPAPLKNDATASCGFSERSSTPHCTMPSGRLSGANAAAADGPGISQLGEVAPLPTL
SAPVMEPLINSEPSTGVTEPLTPHQPNHQPSFLTSPQDLASSPMEEDEQHSEADEPPSDEPLSDDPLSP
AEEKLPHIDEYWSDSEHIFLDANIGGVAIAPAHGSVLIECARRELHATTPVEHPNRNHPTRLSLVFYQHK
NLNKPQHGFELNKIKFEAKEAKNKKMKASEQKDQAANEGPEQSSEVNELNQIPSHKALTLTHDNVVTV
SPYALTHVAGPYNHWVID

Name: **PUFa-TDG-TET1(CD)**

Keys: NLS, PUFa, TDG **TET1(1418-2136), Flag tag**

MIDGGGGSDPKKKRKVDPKKKRKVDPKKKRKVGSTGSRNDGGGGSGGGGSGGGGSGRAGILPPKKKR
KVSRGRSRLLEDFRNNRYPNLQLREIAGHIMEFSQDQHGSRFIQLKLERATPAERQLVFNEILQAAYQLM
VDVFGNYVIQKFFEFGSLEQKLALAERIRGHVLSLALQMYGSRVIEKALEFIPSDQQNEMVRELDGHVLKC
VKDQNGNHVVQKCIECVQPQSLQFIIDAFKGQVFALSTHPYGCRVIQRILEHCLPDQTLPILEELHQHTEQL
VQDQYGNYVIQHVLEHGRPEDKSKIVAEIRGNVLVLSQHKFASNVVEKCVTHASRTERAVLIDEVCTMND

GPHSALYTMMKDQYANYVVQKMIDVAEPGQRKIVMHKIRPHIATLRKYTYGKHILAKLEKYYMKNGVDLG
DPKKKRKVDPKKKRKVGGRGGGSGGGSGGGSGGGSGGGSGGGSLEAENAGSYSLQQAQAFYTFPFQ
QLMAEAPNMAVVNEQQMPEEVPAPAPAQEPVQEAPKGRKRKPRTTEPKQPVEPKKPVESKKSGKSAKS
KEKQEKITDTFKVKRKVDRFNGVSEAELLTKTLPDILTFNLDIVIIGINPGLMAAYKGHHYPGPGNHFWKCL
FMSGLSEVQLNHMDDHTLPGKYGIGFTNMVERTTPGSKDLSSKEFREGGRILVQKLQKYQPRIAVFNGK
CIYEIFSKEVFGVKVKNLEFGLQPHKIPDTETLCYVMPSSSARCAQFPRAQDKVHYYIKLKDLRDQLKGIER
NMDVQEVQYTFDLQLAQEDAKKMAVKEEKYDPGYEAAYGGAYGENPCSSEPCGFSSNGLIESVELRGE
SAFSGIPNGQWMTQSFTDQIPSFSNHCGTQEQEEESHAGRGGGGSGGGGSGGGGSGPA**ELPTCSCL**
DRVIQKDKGPYYTHLGAGPSVAAVREIMENRYGQKGNAIRIEIVVYTGKEGKSSHGCPIAKWVLRRSSD
EEKVLCLVRQRTGHHCPTAVMVVLIMVWDGIPLPMADRLYTELTENLKSYNGHPTDRRCTLNENRTCT
CQGIDPETCGASFSFGCSWSMYFNGCKFGRSPSPRRFRIDPSSPLHEKNLEDNLQSLATRLAPIYKQYA
PVAYQNQVEYENVARECRLGSKEGRPFSGVTACLDFCAHPHRDIHNMNNGSTVVCTLTREDNRSLGVI
PQDEQLHVLPLYKLSDTDEFGSKEGMEAKIKSGAIEVLAPRRKKRTCFTQPVPRSGKKRAAMMTEVLA
HKIRAVEKKPIPRIKRKNNSTTTNNSKPSSLPTLGSNTETVQPEVKSETEPHFILKSSDNTKTYSLMPSAP
HPVKEASPGFSWSPKTASATPAPLKNDATASCGFSERSSTPHCTMPSGRLSGANAAAADGPGISQLG
EVAPLPTLSAPVMEPLINSEPSTGVTEPLTPHQPNHQPSFLTSPQDLASSPMEEDEQHSEADEPPSDEP
LSDDPLSPAEEKLPHIDEYWSDSEHIFLDANIGGVAIAPAHGSVLIECARRELHATTPVEHPNRNHPTRL
SLVFYQHKNLNKPQHGFELNKIKFEAKEAKNKKMKASEQKDQAANEGPEQSSEVNELNQIPSHKALTL
THDNVVTVSPYALTHVAGPYNHWVID

Name: **3xFlag-PUFa-TET1(CD)**

Keys: NLS, PUFa, **TET1(1418-2136), 3xFlag tag**

M**DYKDHDGDYKDHDIDYKDDDDK**IDGGGGSDPKKKRKVDPKKKRKVDPKKKRKVGSTGSRNDGGGGS
GGGGSGGGGSGRAGILPPKKKRKVSRGRSRLLEDFRNNRYPNLQLREIAGHIMEFSQDQHGSRFIQLKL
ERATPAERQLVFNEILQAAYQLMVDVFGNYVIQKFFEFGSLEQKLALAERIRGHVLSLALQMYGSRVIEKA
LEFIPSDQQNEMVRELDGHVLKCVKDQNGNHVVQKCIECVQPQSLQFIIDAFKGQVFALSTHPYGCRVIQ
RILEHCLPDQTLPILEELHQHTEQLVQDQYGNYVIQHVLEHGRPEDKSKIVAEIRGNVLVLSQHKFASNVV
EKCVTHASRTERAVLIDEVCTMNDGPHSALYTMMKDQYANYVVQKMIDVAEPGQRKIVMHKIRPHIATLR
KYTYGKHILAKLEKYYMKNGVDLGDPKKKRKVDPKKKRKVGGRGGGGSGGGGSGGGGSGPA**ELPTCS**
CLDRVIQKDKGPYYTHLGAGPSVAAVREIMENRYGQKGNAIRIEIVVYTGKEGKSSHGCPIAKWVLRRS
SDEEKVLCLVRQRTGHHCPTAVMVVLIMVWDGIPLPMADRLYTELTENLKSYNGHPTDRRCTLNENRT
CTCQGIDPETCGASFSFGCSWSMYFNGCKFGRSPSPRRFRIDPSSPLHEKNLEDNLQSLATRLAPIYKQ
YAPVAYQNQVEYENVARECRLGSKEGRPFSGVTACLDFCAHPHRDIHNMNNGSTVVCTLTREDNRSL
GVIPQDEQLHVLPLYKLSDTDEFGSKEGMEAKIKSGAIEVLAPRRKKRTCFTQPVPRSGKKRAAMMTE
VLAHKIRAVEKKPIPRIKRKNNSTTTNNSKPSSLPTLGSNTETVQPEVKSETEPHFILKSSDNTKTYSLMP
SAPHPVKEASPGFSWSPKTASATPAPLKNDATASCGFSERSSTPHCTMPSGRLSGANAAAADGPGIS
QLGEVAPLPTLSAPVMEPLINSEPSTGVTEPLTPHQPNHQPSFLTSPQDLASSPMEEDEQHSEADEPPS
DEPLSDDPLSPAEEKLPHIDEYWSDSEHIFLDANIGGVAIAPAHGSVLIECARRELHATTPVEHPNRNHP
TRLSLVFYQHKNLNKPQHGFELNKIKFEAKEAKNKKMKASEQKDQAANEGPEQSSEVNELNQIPSHKA
LTLTHDNVVTVSPYALTHVAGPYNHWVID

Name: **3xFlag-hDAGG45-PUFa-TET1(CD)**

Keys: NLS, PUFa, GADD45A, **TET1(1418-2136), 3xFlag tag**

DYKDHDGDYKDHDIDYKDDDDKIDGGGGSSGAATMTLEEFSAGEQKTERMDKVGDALEEVLSKALSQR
TITVGVYEAAKLLNVDPDNVVLCLLAADEDDDRDVALQIHFTLIQAFCCENDINILRVSNPGRLAELLLLETD
AGPAASEGAEQPPDLHCVLVTNPHSSQWKDPALSQLICFCRESRYMDQWVPVINLPERSRTGAATMIDG
GGGSDPKKKRKVDPKKKRKVDPKKKRKVGSTGSRNDGGGGSGGGGSGGGGSGRAGILPPKKKRKVS
RGRSRLLEDFRNNRYPNLQLREIAGHIMEFSQDQHGSRFIQLKLERATPAERQLVFNEILQAAYQLMVDV
FGNYVIQKFFEFGSLEQKLALAERIRGHVLSLALQMYGSRVIEKALEFIPSDQQNEMVRELDGHVLKCVKD
QNGNHVVQKCIECVQPQSLQFIIDAFKGQVFALSTHPYGCRVIQRILEHCLPDQTLPILEELHQHTEQLVQ
DQYGNYVIQHVLEHGRPEDKSKIVAEIRGNVLVLSQHKFASNVVEKCVTHASRTERAVLIDEVCTMNDGP
HSALYTMMKDQYANYVVQKMIDVAEPGQRKIVMHKIRPHIATLRKYTYGKHILAKLEKYYMKNGVDLGDP
KKKRKVDPKKKRKVGGRGGGGSGGGGSGGGGSGPA**ELPTCSCLDRVIQKDKGPYYTHLGAGPSVAA**
VREIMENRYGQKGNAIRIEIVVYTGKEGKSSHGCPIAKWVLRRSSDEEKVLCLVRQRTGHHCPTAVMVV

**LIMVWDGIPLPMADRLYTELTENLKSYNGHPTDRRCTLNENRTCTCQGIDPETCGASFSFGCSWSMYF
NGCKFGRSPSPRRFRIDPSSPLHEKNLEDNLQSLATRLAPIYKQYAPVAYQNQVEYENVARECRLGSK
EGRPFSGVTACLDFCAHPHRDIHNMNNGSTVVCTLTREDNRSLGVIPQDEQLHVLPLYKLSDTDEFGSK
EGMEAKIKSGAIEVLAPRRKKRTCFTQPVPRSGKKRAAMMTEVLAHKIRAVEKKPIPRIKRKNNSTTTN
NSKPSSLPTLGSNTETVQPEVKSETEPHFILKSSDNTKTYSLMPSAPHPVKEASPGFSWSPKTASATPA
PLKNDATASCGFSERSSTPHCTMPSGRLSGANAAAADGPGISQLGEVAPLPTLSAPVMEPLINSEPST
GVTEPLTPHQPNHQPSFLTSPQDLASSPMEEDEQHSEADEPPSDEPLSDDPLSPAEEKLPHIDEYWSD
SEHIFLDANIGGVAIAPAHGSVLIECARRELHATTPVEHPNRNHPTRLSLVFYQHKNLNKPQHGFELNKI
KFEAKEAKNKKMKASEQKDQAANEGPEQSSEVNELNQIPSHKALTLTHDNVVTVSPYALTHVAGPYN
HWV**ID

Supplementary References

1. Wang T, Wei JJ, Sabatini DM, Lander ES. Genetic screens in human cells using the CRISPR-Cas9 system. *Science* **343**, 80-84 (2014).

2. Yusa K, Zhou L, Li MA, Bradley A, Craig NL. A hyperactive piggyBac transposase for mammalian applications. *Proc Natl Acad Sci U S A* **108**, 1531-1536 (2011).

3. Cheng AW*, et al.* Casilio: a versatile CRISPR-Cas9-Pumilio hybrid for gene regulation and genomic labeling. *Cell Res* **26**, 254-257 (2016).

4. Liu XS*, et al.* Editing DNA Methylation in the Mammalian Genome. *Cell* **167**, 233-247 e217 (2016).
