# Supplementary material for: Enhanced CRISPR-based DNA demethylation by Casilio-ME-mediated RNA-guided coupling of methylcytosine oxidation and DNA repair pathways
Source: Nat Commun. 2019 Sep 20;10:4296. doi: 10.1038/s41467-019-12339-7 (PMC6754513; doi:10.1038/s41467-019-12339-7)
Supplement: Supplementary file 2 — Reporting Summary [file 41467_2019_12339_MOESM2_ESM.pdf]

nature research

Corresponding author(s): Albert W. Cheng
Last updated by author(s): Aug 21, 2019

Reporting Summary

Nature Research wishes to improve the reproducibility of the work that we publish. This form provides structure for consistency and transparency in reporting. For further information on Nature Research policies, see Authors & Referees and the Editorial Policy Checklist.

Statistics

For all statistical analyses, confirm that the following items are present in the figure legend, table legend, main text, or Methods section.

n/a	Confirmed	
☐	☒	The exact sample size (*n*) for each experimental group/condition, given as a discrete number and unit of measurement
☐	☒	A statement on whether measurements were taken from distinct samples or whether the same sample was measured repeatedly
☐	☒	The statistical test(s) used AND whether they are one- or two-sided<br>*Only common tests should be described solely by name; describe more complex techniques in the Methods section.*
☒	☐	A description of all covariates tested
☒	☐	A description of any assumptions or corrections, such as tests of normality and adjustment for multiple comparisons
☐	☒	A full description of the statistical parameters including central tendency (e.g. means) or other basic estimates (e.g. regression coefficient) AND variation (e.g. standard deviation) or associated estimates of uncertainty (e.g. confidence intervals)
☐	☒	For null hypothesis testing, the test statistic (e.g. *F*, *t*, *r*) with confidence intervals, effect sizes, degrees of freedom and *P* value noted<br>*Give P values as exact values whenever suitable.*
☒	☐	For Bayesian analysis, information on the choice of priors and Markov chain Monte Carlo settings
☒	☐	For hierarchical and complex designs, identification of the appropriate level for tests and full reporting of outcomes
☒	☐	Estimates of effect sizes (e.g. Cohen's *d*, Pearson's *r*), indicating how they were calculated

Our web collection on statistics for biologists contains articles on many of the points above.

Software and code

Policy information about availability of computer code

Data collection	ViiA7 software version1.2.2 or QuantStudio version 1.3 (Applied Biosystems by Life technologies). Next generation sequencing data was collected by standard Illumina software.
Data analysis	Microsoft Excel, GraphPad Prism 8, ViiA7 software V1.2.2 or QuantStudio version 1.3 (Applied Biosystems by Life technologies), Bismark, methylKit, BiQ Analyzer 3, BiQ Analzer HiMod and DESeq2.

For manuscripts utilizing custom algorithms or software that are central to the research but not yet described in published literature, software must be made available to editors/reviewers. We strongly encourage code deposition in a community repository (e.g. GitHub). See the Nature Research guidelines for submitting code & software for further information.

Data

Policy information about availability of data

All manuscripts must include a data availability statement. This statement should provide the following information, where applicable:

- Accession codes, unique identifiers, or web links for publicly available datasets
- A list of figures that have associated raw data
- A description of any restrictions on data availability

The data that support the findings of this study are included in the published article and in the Supplemental Information, and are available from corresponding author upon reasonable request. Data containing RNAseq and RRBS raw sequencing read files were deposited onto sequencing read archive (SRA) with accession number PRJNA515359. New plasmids will be deposited to Addgene repository.

Field-specific reporting

Please select the one below that is the best fit for your research. If you are not sure, read the appropriate sections before making your selection.

☒ Life sciences ☐ Behavioural & social sciences ☐ Ecological, evolutionary & environmental sciences

For a reference copy of the document with all sections, see nature.com/documents/nr-reporting-summary-flat.pdf

Life sciences study design

All studies must disclose on these points even when the disclosure is negative.

Sample size	No sample size calculation was performed.
Data exclusions	No data was excluded.
Replication	All attempts at replication were successful. The repeat informations for each experiment are stated in the figure legends where it is applicable.
Randomization	The experiments were not randomized.
Blinding	Blinding was not used for the experiments.

Reporting for specific materials, systems and methods

We require information from authors about some types of materials, experimental systems and methods used in many studies. Here, indicate whether each material, system or method listed is relevant to your study. If you are not sure if a list item applies to your research, read the appropriate section before selecting a response.

Materials & experimental systems

n/a	Involved in the study	
☐	☒	Antibodies
☐	☒	Eukaryotic cell lines
☒	☐	Palaeontology
☒	☐	Animals and other organisms
☒	☐	Human research participants
☒	☐	Clinical data

Methods

n/a	Involved in the study	
☒	☐	ChIP-seq
☒	☐	Flow cytometry
☒	☐	MRI-based neuroimaging

Antibodies

Antibodies used	All antibodies used in this study are commercially available antibodies and have been used in previous research.<br>Mouse monoclonal ANTI-FLAG M2 antibody (Sigma cat# F1804); mouse monoclonal CRISPR/Cas9 antibody [7A9] (Epigenteck cat# A-9000-100) and mouse monoclonal anti-actin antibody, clone C4 (sigma cat # MAB 1501).
Validation	Antibodies used were validated by the indicated suppliers and were tested against control samples when applicable.

Eukaryotic cell lines

Policy information about cell lines

Cell line source(s)	HEK293T, U2OS and LNCaP from American Type Culture Collection (ATCC).
Authentication	Cell lines were authenticate by the supplier.
Mycoplasma contamination	No contamination was detected by the supplier.
Commonly misidentified lines<br>(See ICLAC register)	No commonly misidentified cell lines were used.
